# Single‐cell RNA sequencing reveals a mechanism underlying the susceptibility of the left atrial appendage to intracardiac thrombogenesis during atrial fibrillation

**DOI:** 10.1002/ctm2.1297

**Published:** 2023-06-05

**Authors:** Jie Yang, Hu Tan, Mengjia Sun, Renzheng Chen, Zhao Jian, Yuanbin Song, Jihang Zhang, Shizhu Bian, Bo Zhang, Yi Zhang, Xubin Gao, Zhen Chen, Boji Wu, Xiaowei Ye, Hailin Lv, Zhen Liu, Lan Huang

**Affiliations:** ^1^ Institute of Cardiovascular Diseases of PLA the Second Affiliated Hospital Army Medical University (Third Military Medical University) Chongqing China; ^2^ Department of Cardiology the Second Affiliated Hospital Army Medical University (Third Military Medical University) Chongqing China; ^3^ Department of Cardiovascular Surgery the Second Affiliated Hospital Army Medical University (Third Military Medical University) Chongqing China

**Keywords:** ADAMTS1, atrial fibrillation, endocardial endothelial cells, single‐cell RNA‐sequencing, TFPI family, thrombogenesis

## Abstract

**Background:**

Atrial fibrillation (AF) is associated with an increased risk of thrombosis of the left atrial appendage (LAA). However, the molecular mechanisms underlying this site‐specificity remain poorly understood. Here, we present a comparative single‐cell transcriptional profile of paired atrial appendages from patients with AF and illustrate the chamber‐specific properties of the main cell types.

**Methods:**

Single‐cell RNA sequencing analysis of matched atrial appendage samples from three patients with persistent AF was evaluated by 10× genomics. The AF mice model was created using *Tbx5* knockout mice. Validation experiments were performed by glutathione S‐transferase pull‐down assays, coimmunoprecipitation (Co‐IP), cleavage assays and shear stress experiments in vitro.

**Results:**

In LAA, phenotype switching from endothelial cells to fibroblasts and inflammation associated with proinflammatory macrophage infiltration were observed. Importantly, the coagulation cascade is highly enriched in LAA endocardial endothelial cells (EECs), accompanying the up‐regulation of a disintegrin and metalloproteinase with thrombospondin motifs 1 (ADAMTS1) and the down‐regulation of the tissue factor pathway inhibitor (TFPI) and TFPI2. Similar alterations were verified in an AF mouse model (*Tbx5* ^+/−^) and EECs treated with simulated AF shear stress in vitro. Furthermore, we revealed that the cleavage of both TFPI and TFPI2 based on their interaction with ADAMTS1 would lead to loss of anticoagulant activities of EECs.

**Conclusions:**

This study highlights the decrease in the anticoagulant status of EECs in LAA as a potential mechanism underlying the propensity for thrombosis, which may aid the development of anticoagulation therapeutic approaches targeting functionally distinct cell subsets or molecules during AF.

## Graphical abstract

- Single‐cell RNA‐sequencing revealed atrial appendage‐specific transcriptional heterogeneity of atrial fibrillation patients.
- Differences in the local expression of anticoagulants by endocardial endothelial cells (EECs) contribute to the left atrial appendage (LAA)‐specific propensity for thrombogenesis.
- Deficiency of the anticoagulants TFPI and TFPI2 in the EECs of the LAA can be attributed to the proteolytic activity of ADAMTS1 in response to shear stress.

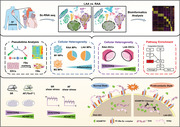

## 1 INTRODUCTION

Atrial fibrillation (AF), characterized by rapid and disorganized electrical activity within the cardiac atria, has become the most prevalent cardiac arrhythmia in recent years.^1^ AF results in the loss of regular contractions and abnormal haemodynamics in the atrium, increasing the risk of developing intracardiac thrombi. In clinical practice, intracardiac thrombus detachment is the primary cause of peripheral artery embolism, especially the life‐threatening intracranial embolism. Autopsy and transesophageal echocardiographic studies have shown that intracardiac thrombi develop predominantly in the left atrial appendage (LAA), whereas few are observed in the right atrial appendage (RAA),^2^, ^3^ highlighting the LAA as a primary target for arterial embolism prevention strategies.

The LAA originates from the embryonic pulmonary veins and the left atrium (LA) and develops into a small ear‐shaped sac that consists of muscular trabeculae.^4^ AF disrupts the contractility of the LAA, resulting in aberrant haemodynamics. The stagnation of blood flow in the LAA alters the turbulence of shear stress, which induces endothelial dysfunction, a potential cause of intracardiac thrombus formation.^5^ Electron microscopy analysis has revealed that endothelial injury increases the risk of clot formation in the LAA of patients with AF.^6^ In both the LAA and RAA, endocardial endothelial cells (EECs), which line the internal surface of the heart chambers, represent a barrier between the myocardium and circulation. EECs provide an antithrombotic interface with circulating blood through the coordinated expression of anticoagulants,^7^, ^8^ including the tissue factor pathway inhibitor (TFPI), which suppresses the tissue factor (TF) coagulation cascade. The balance between circulating TFPI and TF levels is altered in patients with nonvalvular AF and cardiogenic thromboembolism.^9^ Moreover, human endothelial cells (ECs) can respond to uneven shear stress due to altered blood flow by modulating the expression and activity of TFPI.^8^, ^10^ Thus, the differential functioning of the EECs between the LAA and RAA, especially in terms of anticoagulant synthesis, may influence the preferential formation of intracardiac thrombi in the LAA of patients with AF.

Additionally, AF is characterized by chronic inflammation due to immune cell activation and proinflammatory cytokine release,^11^ which promote cardiac fibrosis and structural remodelling of the atrium, including collagen accumulation and amyloid deposition, especially in the LAA.^12^ Although studies have focused on the role of inflammation and fibrosis in AF, the differences between these processes, particularly in terms of cellular taxonomy and molecular regulatory networks, remain unclear. Therefore, our objective was to (1) clarify the cellular composition and transcriptomic heterogeneity within individual cell types in the LAA and RAA of patients with AF; (2) reveal the mechanisms underlying thrombogenesis, inflammation and cardiac fibrosis in patients with AF; and (3) provide a target for developing potential therapeutic approaches for intracardiac thrombi and preventive strategy for stroke in patients with AF.

## 2 MATERIALS AND METHODS

### 2.1 Samples of the human atrial appendage

Atrial appendage samples were obtained from three patients with persistent AF and a history of cardiogenic thromboembolism. The patients had not received warfarin or other antiplatelet drugs, or haemorheological agents, namely analgesics or oestrogen‐containing drugs. The patients underwent a modified maze procedure during open‐heart surgery at Xinqiao Hospital; their clinical data are presented in Table S1. Surgically removed tissue samples were kept in a tissue storage solution (Miltenyi Biotec, Bergisch Gladbach, Germany) on ice until digestion with DNase I (30 U/mL; Worthington, Columbus, OH, USA), collagenase IV (195 U/mL; Worthington) and collagenase I (10 U/mL; Worthington) in 30% FBS (HyClone, Logan, UT, USA) for 1 h at 37°C with agitation to obtain single‐cell suspensions. The suspensions were centrifuged at 300 × *g* to obtain cell pellets, which were lysed in red blood cell lysis buffer (00‐4333‐57, eBioscience, Waltham, MA, USA), resuspended in RPMI‐1640 medium (11875101, Thermo Fisher Scientific, Waltham, MA, USA) and filtered through a 35 μm cell strainer.

### 2.2 ScRNA‐seq using 10× genomics

Cell suspension (500–1000 living cells/μL; determined using Count Star) was loaded onto a Chromium Single Cell Controller (10× Genomics, Pleasanton, CA, USA) to generate single‐cell gel beads‐in‐emulsion (GEMs) using the Single Cell 3′ Library and Gel Bead Kit V3 (1000075, 10× Genomics). Cells captured were lysed, and the released RNA was barcoded by reverse transcription in individual GEMs using an Agilent 4200 TapeStation system (Agilent Technologies Inc., Santa Clara, CA, USA) under the following conditions: 53°C for 45 min, followed by 85°C for 5 min and then left at 4°C. cDNA was generated (CapitalBio), and single‐cell RNA sequencing (scRNA‐seq) libraries were prepared using the Single Cell 3′ Library Gel Bead Kit V3 according to the manufacturer's instructions and sequenced on an Illumina NovaSeq X (Illumina Inc., San Diego, CA, USA) instrument with a sequencing depth of at least 100 000 reads per cell and 150 bp reads at the paired end (CapitalBio). All sequencing data were submitted to the National Center for Biotechnology Information Gene Expression Omnibus (NCBI GEO) database (Submission ID: SUB11167988 and SUB11200864; BioProject ID: PRJNA815461).

### 2.3 ScRNA‐seq data processing

Raw data from the three samples were processed using the Cell Ranger Software Suite (version 3.0.1) (10× Genomics, Pleasanton, CA, USA)^13^ with default mapping parameters to aggregate the three‐cell ranger count runs and remove the batch effect.^14^ Data quality control, visualization and analysis were performed using CapitalBio with the Seurat R package (v3.3.2). Cells expressing <800 or >4000 genes per cell, and those with mitochondrial gene percentages >.1 were removed. Cells with mitochondrial unique molecular identifier (UMI) counts >6% or ribosomal UMI counts >50% were also considered abnormal. The batch effect was corrected, and the merged object was integrated by running Harmony (version 1.0). The top 30 Harmony dimensions were provided as input for Uniform Manifold Approximation and Projection (UMAP), which visualized the first two dimensions at a clustering resolution of .6. All steps were performed using functions implemented in the Harmony and Seurat packages (NormalizeData, FindVariableFeatures, ScaleData, RunPCA, FindNeighbours, FindClusters and RunUMAP) with default parameters. Finally, 32 326 single cells were analysed. Cell types were identified using CellMarker. Data visualization in two dimensions was performed using *t*‐distributed stochastic neighbour embedding (*t*‐SNE) clustering analysis.

To process and analyse the scRNA‐seq data from public resources, we first acquired raw sequencing reads at the Broad Institute Single Cell Portal (https://portals.broadinstitute.org/single_cell). Raw scRNA‐seq data from specific samples were extracted, realigned and analysed by NovelBio Co., Ltd. using the NovelBrain Cloud Analysis Platform (www.novelbrain.com). We applied fastp^15^ with the same parameter filtering the adaptor sequence and removed low‐quality reads to yield clean data. Principal component analysis was performed based on scaled data with the top 2000 variable genes and the top 10 principal components used for *t*‐SNE and UMAP construction. Using a graph‐based cluster method, we acquired the results of the unsupervised cell cluster for the top 10 principal components. We calculated marker genes using the FindAllMarkers function with the Wilcoxon rank sum test algorithm, according to the following criteria: log_2_ (fold change) ≥1.2; *p* value <.05; min.pct >.1. To identify the detailed cell type, clusters of the same cell type were selected for revised *t*‐SNE analysis, graph‐based clustering and marker analysis.

### 2.4 Identification of differentially expressed genes

Differentially expressed genes (DEGs) in each cell group were detected using the FindMarkers function in Seurat with the default parameter ‘test.use = wilcox’, and the false discovery rate was estimated using the Benjamini–Hochberg method. DEGs were filtered using log_2_ (fold change) ≥1.2 and *p*‐value <.05 as default parameters. The significant DEGs were used to construct heat map plots.

### 2.5 Gene ontology and Kyoto Encyclopedia of Genes and Genomes enrichment analysis

Significant DEGs were subjected to pathway enrichment analysis of the gene ontology (GO) and Kyoto Encyclopedia of Genes and Genomes (KEGG) using the R package cluster Profiler. The top 20 enriched GO terms (*p* < .05) were visualized using the enrichplot R package.^16^ The functional enrichment analysis of DEGs was conducted using the Metascape webtool (www.metascape.org). The scRNA‐seq data set and data on DEGs and relevant cell‐type‐specific marker genes are available in Table S2.

### 2.6 Pseudotime trajectory analysis

Monocle2 (v 2.14.0)^17^ was used to construct the pseudotime trajectory of the selected cell populations. The top 1100 DEGs (*p*‐value <.01) identified by Seurat were selected as order genes to sort cells in pseudotime order. Using the ‘orderCells’ function, we recognized cells at the start point of the pseudotime and set this state as the ‘root_state’ argument. The discriminative dimensionality reduction with trees method was applied to reduce data dimensions and construct the consequent development paths. The DEGs in pseudotime were calculated using the function ‘differential Gene Test’ (*p* < 10^−20^) and visualized using ‘plot_cell_trajectory’. Genes selected with smoothed scaled expression were plotted using the function ‘plot_pseudotime_heatmap’.

### 2.7 Gene co‐expression network

Genetic co‐expression network analysis was performed to identify the relationship and correlation between genes across the samples. The average gene expression profile of a sample was calculated using the Seurat average expression function (v3.2.0). The Pearson correlation coefficient of the gene was then calculated. The network file was imported into Cytoscape (v3.4.0) and visualized based on the degree of topological properties of this network.

### 2.8 SCENIC transcription factor analysis

The raw count matrix of EECs was used in single‐cell regulatory network inference and clustering (SCENIC).^18^ To identify gene regulatory networks, activated regulons were analysed using pyscenic (v0.10.3) with raw count matrix as input. The co‐expression network was calculated by the runGenie3 function, and the regulons were identified by the RcisTarget package. The regulon activity for each cell was scored by the AUCell algorithm.

### 2.9 Calculation of cell type similarity

For the calculation of cell type similarity, variable genes were selected using the FindVariableGenes function of Seurat (v3.2.0). The average gene expression profile of a cell type was then calculated using variable genes. The Pearson correlation coefficient for each cell type was calculated.

### 2.10 Experimental AF mouse model

The AF mouse model was created using knockout mice of the *Tbx5* locus with a background of C57/B6J as recently reported.^19^ Because homozygous null mice are growth‐arrested and die from severe heart defects, floxed mice with conditional deletion of T‐box transcription factor 5 (*Tbx5^fl/fl^*) were crossed with CMV‐Cre mice to reproduce the *Tbx5^fl/+^*; CMV‐Cre mice. *Tbx5^fl^* mice were created using the CRISPR/Cas9 system at Cyagen Biosciences (Santa Clara, CA, USA). Briefly, mixtures of Cas9 protein (M0646M, NEB), sgRNA (Cyagen Biosciences) and donor vector containing loxP sites were injected into fertilized mouse eggs. CMV‐Cre mice (#006054) were obtained from The Jackson Laboratory and crossed with *Tbx5^fl/fl^* mice to reproduce heterozygote mice for both alleles (*Tbx5^fl/+^*; CMV‐Cre). Sequences between loxP sites were ablated to reduce *Tbx5* expression. CMV‐Cre mice of similar age were used as sinus rhythm (SR) controls. Mice were anesthetized, and the onset and duration of AF (f waves) or atrial flutter (F waves) were determined using echocardiography. The absence of P waves, irregular heartbeats and R–R intervals in the *Tbx5^fl/+^*; CMV‐Cre mice confirmed the successful establishment of the AF mouse model.

### 2.11 TFPI/ TFPI2 cleavage assays

Reaction mixtures (30 μL) containing a disintegrin and metalloproteinase with thrombospondin motifs 1 (ADAMTS1) (1 μg) and TFPI (2 μg) or TFPI2 (2 μg) and in reaction buffer (50 mM Tris–HCl [pH 7.4], .05% Brij‐35 [A610217‐0250; Sangon Biotech], 5 mM CaCl_2_ [C128339; Aladdin], 150 mM NaCl [S196955; Aladdin], 1 μM leupeptin [L111765; Aladdin], 1 μM pepstatin [P113168; Aladdin] and pefabloc [AEBSF, A301913; Aladdin]) were incubated for 5, 15, 30, 60, 120 or 180 min at 37°C. The recombinant proteins above were expressed by the *Escherichia coli* BL21 cell system with a purity >90%, provided by GeneCreate Biological Engineering Co. Ltd (Wuhan, China). Reactions were stopped with SDS loading buffer, and samples were resolved using 12% SDS–PAGE gels, which were then stained with Coomassie Brilliant Blue (P0017; Beyotime Biotechnology) and analysed using scanning densitometry with a Gilford spectrophotometer at 560 nm.

### 2.12 Measurement of TFPI and TFPI2 activity

Recombinant TFPI or TFPI2 was incubated with ADAMTS1, as mentioned above, and the inhibition of factor Xa (FXa) activity was determined as previously described.^20^ This assay is based on the abilities of TFPI or TFPI2 in the sample, in the presence of FXa, to inhibit the catalytic activity of TF/FVIIa. Briefly, after incubation for the indicated time in the above cleavage assays, recombinant fragments cleaved with TFPI/TFPI2 or TFPI/TFPI2 were prepared and mixed with recombinant FXa (1 nM; ab92703; Abcam), recombinant TF (1/400 vol/vol), recombinant FVIIa (10 nM) in 50 mM TBS (pH 7.4), 100 mM NaCl, 5 mM CaCl_2_, .1% BSA and 5 mM EDTA at RT. The reaction was initiated by adding FXa (.2 nmol/L), and the residual catalytic activity of TF/FVIIa was detected using a chromogenic assay and continuously monitored at 405 nm for 180 min.

### 2.13 Other molecular biology exeperiments

Histological analysis, isolation and treatment of primary mouse EECs, EECs purification, western blotting, immunohistochemistry, immunofluorescence, real‐time reverse transcription‐PCR, coimmunoprecipitation, glutathione S‐transferase pulldown assays, rigid‐body docking and shear stress experiments were shown in the supplementary material.

## 3 RESULTS

### 3.1 Cellular landscape of the atrial appendages in patients with AF

**FIGURE 1:**
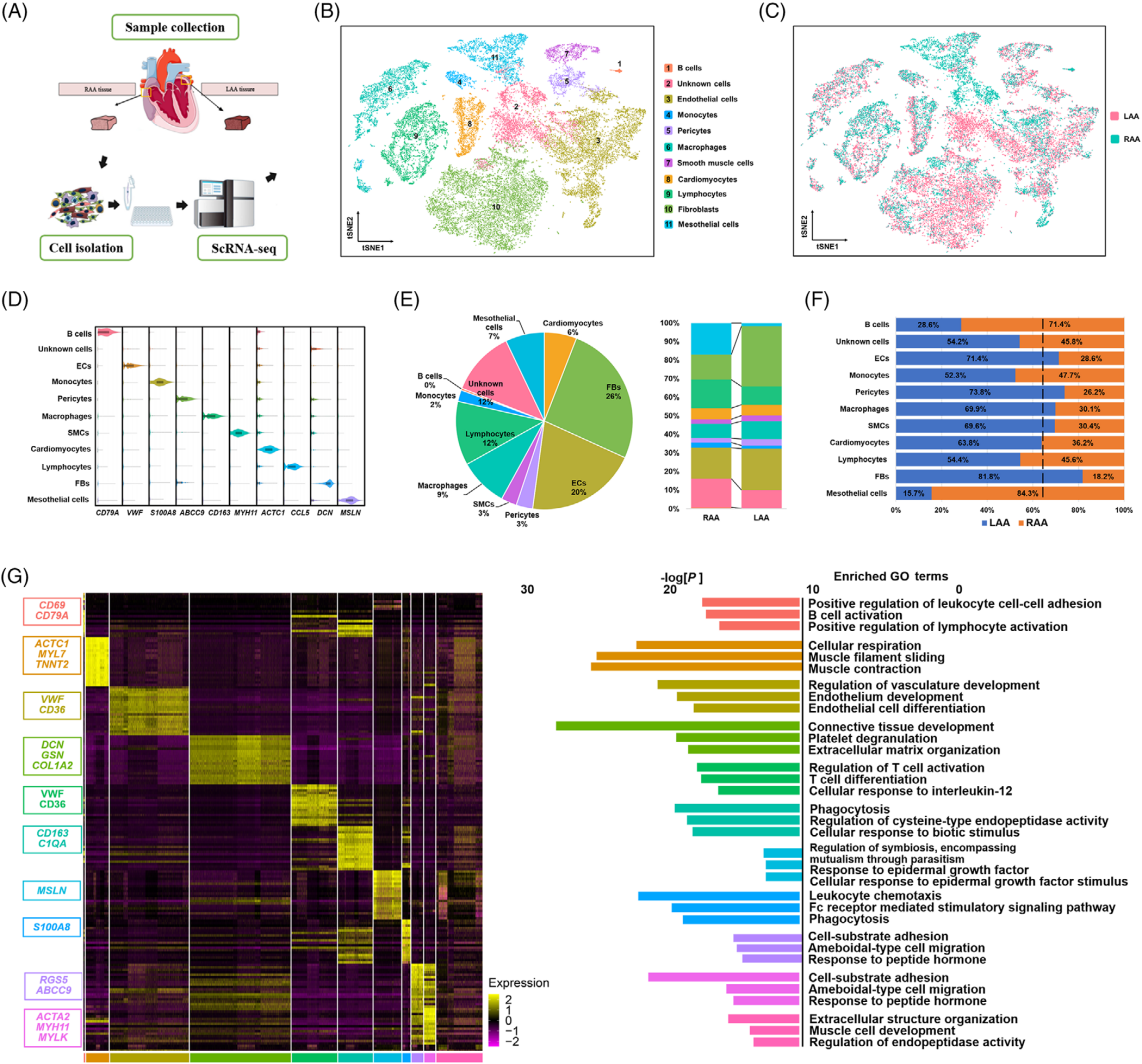
Gene expression and cell atlas of atrial appendage samples from patients with atrial fibrillation. (A) Schematic representation of the single‐cell study process. Samples obtained from the left atrial appendage (LAA) (*n* = 3) and right atrial appendage (RAA) (*n* = 3) of three patients were processed using Chromium 10× chemistry and analysed using single‐cell RNA‐sequencing (scRNA‐seq). (B and C) Visualization of *t*‐distributed stochastic neighbour embedding (*t*‐SNE) of 32 326 cells and 11 major cardiac cell types. Each dot represents a single cell and is coloured according to the cell type designation (B) and sample source (C). The numbers correspond to cell cluster labels. (D) Violin plots of the expression of specific marker genes in each cell type. (E) Distribution of the identified cells according to cell type (left) and their proportions in the LAA and RAA (right). The colour code is shown in (B). (F) Distribution of each cell type between the LAA and RAA. The dashed vertical line indicates the expected proportions (the total number of LAA cells divided by the total number of cells from all specimens). (G) Left: Heat map showing the 20 most up‐regulated genes in each cell type; cell populations were identified based on the expression of the indicated marker genes. Right: gene ontology (GO) enrichment analysis using all genes that reached an average log_2_ fold change ≥ 1.2 for the given cell cluster. The top three GO terms are presented for each cluster. CM, cardiomyocyte; EC, endothelial cell; FB, fibroblast; MP, macrophage; SMC, smooth muscle cell.

Paired LAA and RAA tissues were collected from three patients with persistent AF who underwent open‐heart surgery, digested enzymatically and analysed for single‐cell gene expression (Figure 1A). We observed the distribution and correction of genes with UMIs in 44 759 cells (Figure S1A–C), representing the transcript of doublets or multiplets within an acceptable range. Filtering cells according to the number of genes expressed resulted in 32 326 cells with 1749 genes per cell. The median number of UMIs was 6146, and the average proportion of transcript counts derived from mitochondria‐encoded genes was 6.62% (Figure S1D–F). All high‐quality cells were integrated into a comparable unbatched data set and subjected to principal component analysis after correcting for read depth and mitochondrial read counts.

The hierarchical clustering of the 32 326 cardiac cells using *t*‐SNE (Figure S2A) revealed a high correlation among the 30 obtained clusters (Figure 1B), allowing the classification of individual cells into types of homogeneous states. Sorting the data according to the differential expression of established lineage markers revealed that the cardiac cell population contained 11 major cell types (Figure 1B). Five clusters (1, 4, 6, 8 and 9) were separated from a more continuous group consisting of clusters 3, 5, 7, 10 and 11 centred on cluster 2. Each cluster expressed distinct signature genes and consisted of cells from both atrial appendages (Figure 1C), suggesting robust reproducibility without noticeable batch effects. We identified 1911 cardiomyocytes (expressing *ACTC1*, *MYL7* and *TNNT2*), 8369 fibroblasts (FBs; expressing *DCN*, *GSN* and *COL1A2*), 6565 ECs (expressing *VWF* and *CD36*), 975 pericytes (PCs) (expressing *RGS5* and *ABCC9*), 922 smooth muscle cells (SMCs; expressing *ACTA2*, *MYH11* and *MYLK*), 2878 macrophages (expressing *CD163* and *C1QA*), 3773 lymphocytes (expressing *NKG7*, *CCL5* and *IL7R*), 679 monocytes (expressing *S100A8*), 119 B cells (expressing *CD69* and *CD79A*) and 2300 mesothelial cells (expressing *MSLN*) (Figure 1D; Table S2). A group of unidentified cells not expressing lineage‐specific markers were named unknown cells. In general, ECs, FBs and macrophages were the groups most represented (Figure 1E), with a differential distribution in the two appendages (Figure 1F and Figure S3A) reflecting the true distinctions between the LAA and RAA. The top 20 DEGs for each cell type were analysed for term enrichment of the GO (Figure 1G and Figures S8–S11). The heterogeneity within the cell types is described in detail later; data for other cell types are presented in Figures S8–S11.

### 3.2 ECs in atrial appendages

**FIGURE 2:**
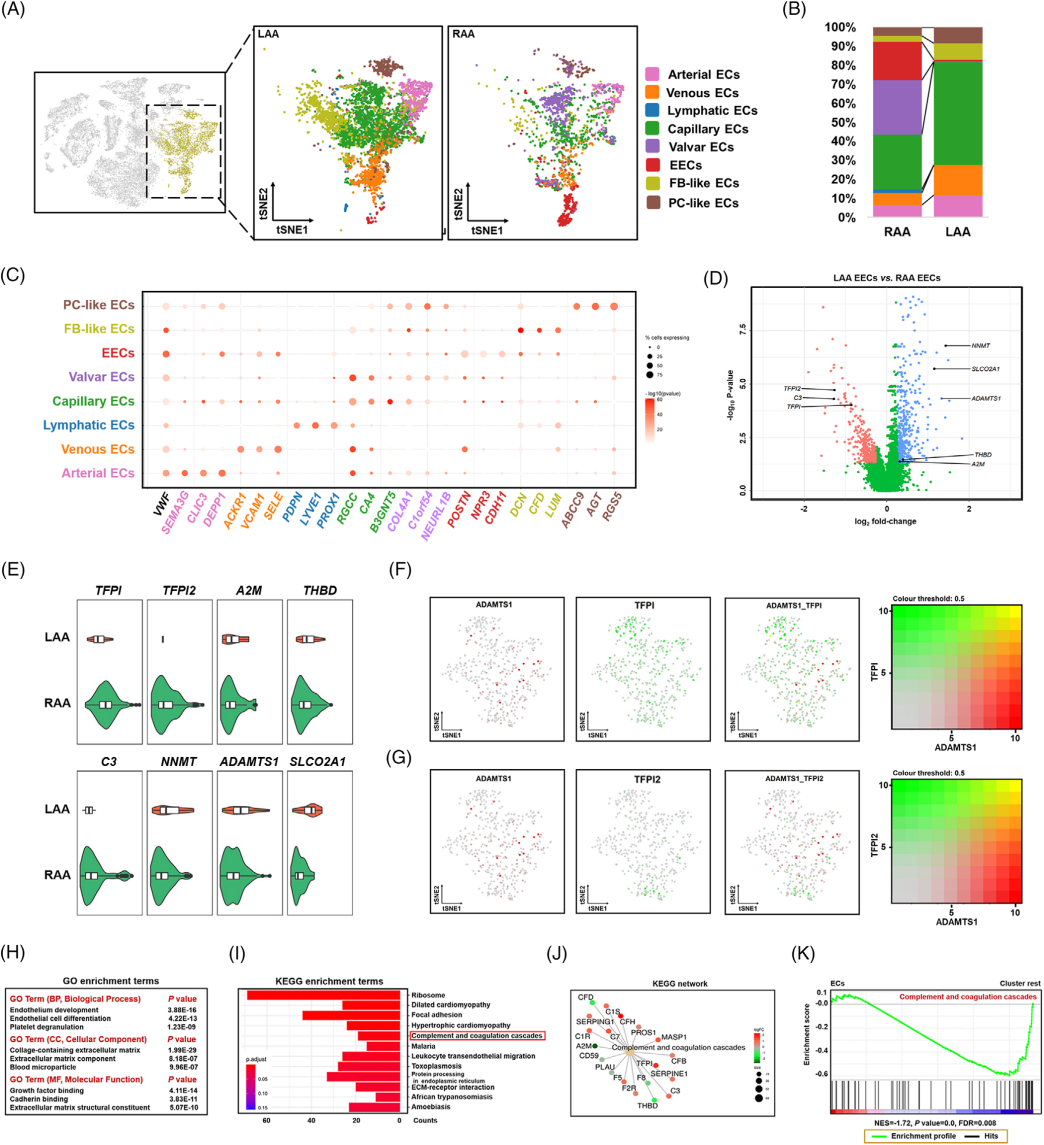
Subpopulations of endothelial cells and characteristics of the atrial appendages of patients with atrial fibrillation (AF). (A) *t*‐Distributed stochastic neighbour embedding (*t*‐SNE) plots showing eight endothelial cell (EC) subclusters in the left atrial appendage (LAA) and right atrial appendage (RAA). Each dot represents an individual cell. (B) Proportions of the eight EC subclusters in the LAA and RAA. The colour code is shown in (A). (C) Dot plot shows the percentage of cells that express the indicated marker genes. The size of the dot corresponds to the proportion of cells detected and the intensity of the colour, to the expression (mean log_10_ *p*‐value). (D) Volcano plot illustrating differential gene expression in endocardial endothelial cells (EECs) from the LAA and RAA. Genes down‐regulated and up‐regulated in the EECs of the LAA are coloured pink and blue, respectively. (E) Violin plots showing the expression of genes up‐regulated in the RAA (*TFPI*, *TFPI2*, *A2M* and *THBD*) and LAA (*C3*, *NNMT*, *ADAMTS1* and *SLCO2A1*). Boxes indicate the lower and upper quartiles (from left to right), the line within each box indicates the median value, and the whiskers show 1.5× interquartile range (IQR). (F) *t*‐SNE plots are coloured according to the expression of a disintegrin and metalloproteinase with thrombospondin motifs 1 (ADAMTS1) (red) and tissue factor pathway inhibitor (TFPI) (green) in EECs. (G) *t*‐SNE plots are coloured according to the expression of ADAMTS1 (red) and tissue factor pathway inhibitor 2 (TFPI2) (green) in the EECs. (H) Top categories of differentially expressed genes (DEGs) up‐regulated in the LAA based on gene ontology (GO) enrichment analysis. (I) Top 10 significant Kyoto Encyclopedia of Genes and Genomes (KEGG) pathways enriched in DEGs up‐regulated in the LAA. The horizontal axis shows the −log_10_ *p*‐value. (J) The KEGG network shows 19 genes assigned to the coagulation cascade. The size of the point indicates the total number of genes assigned, and the colour reflects the *p*‐value correlation. (K) Gene set enrichment analysis (GSEA) results showing the enrichment of EC in complement and coagulation cascades compared to other cell types.

The initial stage of thrombogenesis is characterized by abnormal haemodynamics and EC dysfunction. Therefore, in this study, we focused on the local status of the ECs in each appendage. As ECs were more abundant in the LAA than in the RAA (71.4% vs. 28.6%; Figure 1F), we investigated the composition of the EC subtypes in the appendages using second‐level clustering (Figure 2A and Figure S4A–C). According to expression profiles that reflect the functional commitment of cells, eight subtypes were identified in both appendages. However, their distribution differed significantly between the LAA and the RAA (Figure 2B and Figure S3B). The clustering identified genes specific for the EC subtypes^21^, ^22^, ^23^ (viz. *SEMA3G* and *CLIC3* in arterial ECs, *ARCKR1* and *VCAM* in venous ECs, *PROX1* and *PDPN* in lymphatic ECs, and *NPR3* and *CDH11* in EECs), along with the ubiquitous expression of *VWF* (Figure 2C). Furthermore, FB‐like ECs expressed typical FB markers, including *DCN* and *CFD*, and ECs similar to PCs strongly expressed *ABCC9* and *AGT*. The EEC subpopulation was considerably lower in the LAA than in the RAA (.90% vs. 20.27%, Figure 2B); the high levels of EECs of the RAA observed in patients suggested an effect beyond individual variability (Figure S4E). EECs, which form the innermost layer of the heart wall, are directly exposed to blood turbulence and develop progressive structural defects during AF due to LA dilatation. Of 790 significant DEGs identified in the EEC population (log_2_ fold change ≥ 1.2), 465 and 325 were up‐regulated in the LAA and RAA, respectively (*p* < .05) (Figure 2D). After excluding DEGs without universal expression patterns in the three biological samples, the DEGs most significantly up‐regulated in the LAA were *CCN2*, *MEIS2*, *FABP4*, *NNMT*, *CD55*, *ADAMST1* and *SLCO2A1*, whereas those in the RAA were *TFPI*, *CP*, *C11orf96*, *CCL2*, *CCL14*, *C3* and *TFPI2* (Figure 2E). To further explore the relationship between TFPI/TFPI2 and ADAMTS1 expression, we plotted their relative expression in individual EECs (Figure 2F,G). Almost no cells retained high expression of TFPI/TFPI2 while enriched in ADAMTS1 expression (yellow dots in Figure 2F for TFPI and in Figure 2G for TFPI2). In general, TFPI and TFPI2 are expressed in EECs (green dots in Figure 2F,G), and most EECs expressing ADAMTS1 (red dots in Figure 2F,G) have low or undetectable levels of TFPI or TFPI2.

To identify signalling pathways involved in the dysfunction of the EECs related to AF, we performed gene set enrichment analysis and the GO and KEGG analyses of DEGs in the LAA versus RAA (log_2_ fold change ≥ 1.2). Compared to EECs in the RAA, those in the LAA were functionally enriched in biological processes related to endothelium development, EC differentiation, platelet degranulation and in cellular components associated with the extracellular matrix (ECM) and blood microparticles (Figure 2H). According to the KEGG analysis, EECs in the RAA showed the activation of complement and coagulation cascades, including *TFPI* (Figure 2I,J). Activation of the complement and coagulation cascade was also detected in the total population of ECs but not in other cells (Figure 2K), suggesting that the balance between coagulation and anticoagulation in ECs, especially EECs, may be controlled by the expression of *TFPI* and *TFPI2* encoding coagulation inhibitors. These data explain the traditional view that the LAA is more susceptible to intracardiac thrombus formation, which may, at least in part, be attributed to the prothrombotic phenotype of EECs in the LAA of patients with AF. The chamber‐specific gene expression pattern of the EEC subgroups suggested that gene expression may be governed by various upstream regulatory mechanisms. SCENIC analysis was performed to determine the potential transcription factors that drive the difference in prothrombotic state. Active regulon modules (transcription factors together with their target genes) that may be important for the regulation of the coagulation cascade during AF were identified by comparing EECs from two chambers. Calculating the regulation specificity score (RSS) showed that transcription factors, including POU3F1, have the highest transcription activity compared to EECs in the LAA, and in this cell cluster, GLIS3, PRDM1, HOXA1 and WT1 also showed stronger activity (Figure S5A). For EECs that form the RAA, regulon analysis predicted chamber‐specific signatures of EECs associated with ELK4, ELK3, E2F4, SP3 and RELA, in addition to their downstream genes being otherwise expressed more broadly in the RAA cluster (Figure S5B). This was particularly notable for ELK3, which has been confirmed to be involved in the suppression of PAI‐1 expression,^24^ with the highest expression of its regulon (RSS = .805) associated with EECs in the RAA, indicating that transcriptional changes culminate in a terminal antithrombotic state in the RAA. Collectively, these results suggested that the functional diversity of EECs and related transcription factors may act as a regulator for the susceptibility to the thrombogenesis of the LAA.

To better understand the role of AF in the two atria, control cardiac data were taken from the Broad Institute Single Cell Portal (ID SCP498) based on three healthy male individuals (Figure S6A–C).^25^ Reclustering ECs led to the identification of EECs, which showed a high expression of well‐defined endocardium‐specific genes: *NPR3*, *CDH11* ^25^ and *SMOC1* ^26^ (Figure S6D). In addition to the known marker genes of EECs, the similarity of the identified EECs between the present scRNA‐seq data and the referenced public data was also observed after the hierarchical clustering of the most expressed genes (Figure S6E). A Pearson correlation coefficient matrix was calculated for the top 10 variable genes and clustered hierarchically. For *MGP*, *FOS* and *FTL*, data from two databases clustered side by side on both the *x* and *y* axes. The same gene from a different database displayed the highest correlation of gene expression levels (*r* = .927). Such a cross‐specifies correlation identified the similar gene expression traits of EECs between two databases. We screened 1429 DEGs in the EECs, of which 805 were up‐regulated, and 624 were down‐regulated when comparing the LA and RA. Unlike chamber‐specific expression programmes observed in EECs of AF patients, no differential expression signature of *ADAMTS1*, *TFPI* or *TFPI2* was observed between the LA and RA in healthy humans (Figure S6F). High DEG expression was associated with focal adhesion and multiple cardiomyopathies, but no differences were observed in the enrichment of the pathways related to anticoagulant and procoagulant activities (Figure S6G). These results indicated that changes in EECs were largely attributed to AF.

### 3.3 FBs in atrial appendages

**FIGURE 3:**
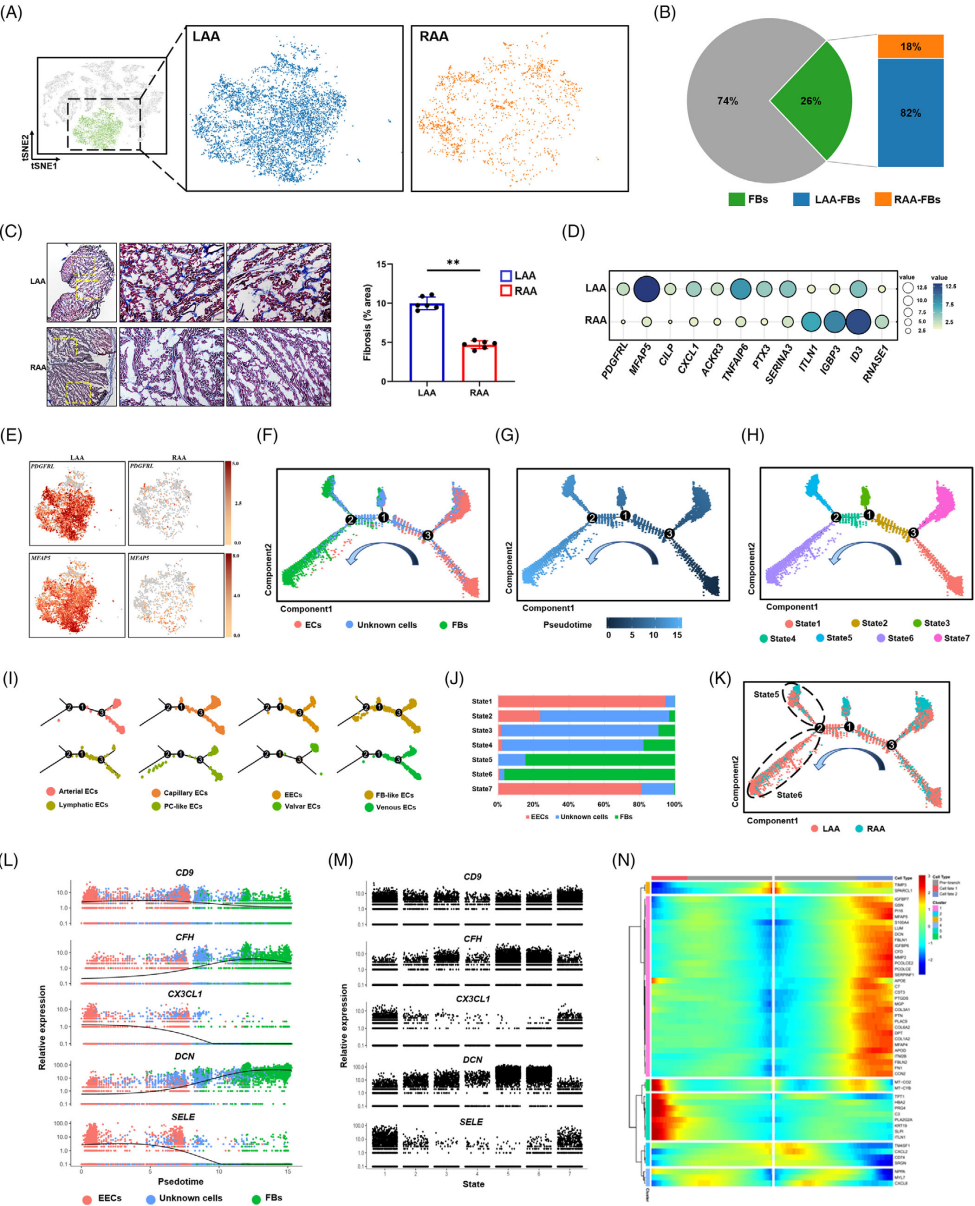
Fibroblast (FB) distribution and transformation in the atrial appendages of patients with atrial fibrillation (AF). (A) *t*‐Distributed stochastic neighbour embedding (*t*‐SNE) plots showing FBs in the left atrial appendage (LAA) and right atrial appendage (RAA). Each dot represents an individual cell. (B) Percentage of FB among identified cells (left) and their distribution between the LAA and RAA (right). (C) Cardiac fibrosis in the LAA and RAA analysed using Masson trichrome staining (*n* = 3 per group); images to the left show boxed areas at higher magnification. Scale bars: 2 mm (left panels) and 100 μm (middle and right panels). The graph shows the percentages of fibrotic areas in each appendage. (D) Relative expression of differentially expressed genes (DEGs) up‐regulated in FBs of the LAA and RAA. Dot size and colour intensity correspond to the expression level. (E) *t*‐SNE plots are coloured according to the expression of marker genes for activated FBs in cells from the LAA and RAA. (F–H) Pseudotemporal order of endocardial endothelial cells (EECs), unknown cells and FBs presented according to cell clusters (F), pseudotime (G) and cell state (H). (I) Split view of the trajectory analysis of endothelial cell (EC) subclusters. (J) Bar graph showing the distribution of the cell type (EECs, unknown cells and FBs) in each cell state (States 1−7). (K) Distribution of cells in the trajectory analysis according to cell source. (L) Expression trajectories of selected genes in pseudotime ordering among cell clusters (ECs, unknown cells and FB). (M) Expression trajectories of selected genes in pseudotime ordering along cell states (States 1−7). (N) Heat map showing the dynamic expression of the indicated genes according to cell type and cluster. The *x*‐axis represents cell type according to pseudotime (from left to right), and the *y*‐axis shows cell clusters; the colour scale indicates the level of gene expression.

In a healthy human heart, FBs are roughly equivalent to or less than cardiomyocytes, especially in the ventricles (Figure S6B).^25^, ^26^ However, in patients with AF, we observed a larger population of FBs than of cardiomyocytes (26% vs. 6%) (Figure 1E). Although this could be partially attributed to technical difficulties in capturing cardiomyocytes in droplets, the AF‐induced pathological changes cannot be ignored. To explore gene expression patterns in the FBs based on the appendage, we analysed the FB distribution between the LAA and RAA (Figure 3A). Most FBs (82% vs. 18%) were present in the LAA (Figure 3B), indicating their possible contribution to fibrotic remodelling in the LAA of a fibrillating heart. In fact, Figure 3C shows a higher level of fibrosis in the LAA than in the RAA of patients with AF, evidenced by the increased collagen content detected using Masson trichrome staining. In the LAA, the FBs predominantly expressed *PDGFRL*,^27^ *MFAP5* ^28^ and *CILP* ^29^, ^30^ (Figure 3D), consistent with the remodelling of the ECM due to profibrotic responses. However, RAA FBs mainly expressed metabolism and proliferation‐related genes, namely *ITLN1*,^31^ *IGFBP3* ^32^ and *RNASE1*,^33^ developed due to their protective functions in ischemic and inflammatory cardiovascular diseases. Other DEGs were related to inflammatory chemokines (CXCL1 and *ACKR3* ^34^) and mesenchymal transformation (*MFAP5*,^28^ *TNFAIP6* ^35^ and *PTX3* ^35^) (Figure 3D,E). These findings suggest the involvement of FBs in tissue remodelling in the LAA and the possibility that they originate from other cell types.

Clustering revealed an undefined population of cells, which may be in a transition state during intercellular transformation. Thus, we constructed a pseudotime trajectory for FBs, ECs and unknown cells. According to pseudotime values calculated based on the expression profile of highly variable genes, we identified seven cell states (Figure 3H) and two cell fates (Figure 3N) and specified the direction of cell transformation. As shown in Figure 3F–H, ECs and FBs were abundant at the two distinct ends of the trajectory, whereas unknown cells and some ECs were found in the middle. The initial set of ECs belonged mainly to states 1 and 7 (Figure 3J). They could re‐differentiate towards unknown cells (after branch point 3, Figure 3F) and FBs (after branch point 2, Figure 3F), representing a continuous transformation state from ECs to unknown cells and then to FBs. Peculiarly, different subgroups of ECs were found in different time periods: endocardial, lymphatic and venous ECs appeared primarily in the middle part closer to the branch point, whereas ECs similar to FBs and PCs were observed mainly in the final phase (Figure 3I), suggesting that they are more likely to respond to fibrosis. After branch point 2, the cells bifurcated towards state 5 in one direction and state 6 in another (Figure 3H). Both contained mostly FBs (Figure 3J) but showed imbalanced cell distribution when grouped with the chamber source (Figure 3K), suggesting that branch point 2 corresponds to the successful induction of transforming the FB phenotype, and that different cell fates existed between the LAA and RAA. Initially (states 1 and 7), cells expressed high levels of inflammatory genes, namely *CX3CL1* and *SELE*, whereas *DCN* was strongly expressed at the end of cell transition (states 5 and 6), and *CFH* was gradually up‐regulated and then slightly down‐regulated (Figure 3L,M), which encoded the activation of complement factor‐related proteins.^36^ Furthermore, *S100A4* and *MFAP5*,^28^ indicative of EndMT, and the ECM‐related markers *DCN*, *COL1A2* and *COL3A1* were significantly up‐regulated in gene module 1 (Figure 3N), suggesting that the FBs of the atrial appendage tissue are derived from ECs that undergo EndMT, particularly EECs of the LAA. These results provide a better understanding of atrial fibrosis, cell fate and functional gene expression changes in EC subtypes residing in the atrial appendages of patients with AF.

### 3.4 Unknown cells in the atrial appendages

To elucidate the functions of the unknown cells in transforming the phenotype, a closer inspection of the indicated subclusters (R1–R5) revealed their signature genes and functional diversity (Figure S7A,B). Expression profiles of unknown meso‐like cells (R1) showed the abundance of *PLA2G2A*, *PRG4* and *ITLN*, which were involved in the process of epicardial fat fibrosis.^37^ The distinguished classical mature cell markers among these clusters also provided evidence for identifying the following specific subtypes. For example, endo‐like cells (R2) highly expressed RGCC, FABP5, CD36 and FABP4, whereas fibro‐like cells (R4) appeared to be more closely related to genes involved in ECM remodelling, associated with *DCN*, *LUM*, *CFD* and *MFAP4*, suggesting their different roles in EndMT processes. Compared to other unknown cells, mono‐like cells (R3) showed a prominent enrichment in inflammatory and immunity processes, consistent with a relatively higher expression of chemokines and genes related to chemokine receptors (Figure S7A,B). The remaining unknown cells (R5) displayed greater involvement in the regulation of nitric oxide and immune processes (Figure S7A,B), as well as higher expression levels of secreted and membrane genes compared to their other unknown cell counterparts (Figure S7A,B). Based on the pseudotime analysis, endo‐ and fibro‐like cells appear more congregated in states 5 and 6, whereas meso‐ and mono‐like cells contribute more to the branching parts (Figure S7C). These results indicate a functional overlap between unknown cells and other cell types in the adult heart, supporting their major function in the transition state and emphasizing their additional pseudotime‐specific involvement in EndMT.

### 3.5 Immune cells in the atrial appendages

**FIGURE 4:**
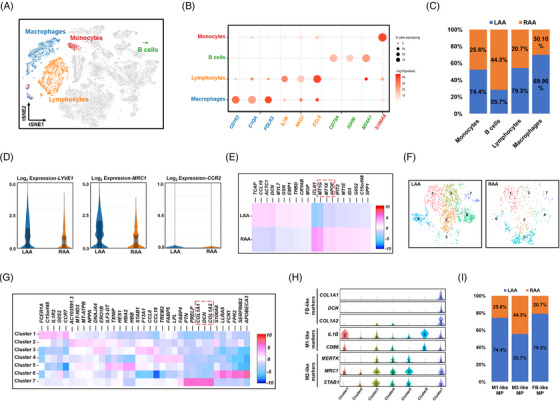
Classification and characteristics in the atrial appendages of patients with atrial fibrillation (AF). (A) *t*‐Distributed stochastic neighbour embedding (*t*‐SNE) plot of the indicated immune cell subclusters superimposed on the global *t*‐SNE plot. Each dot represents an individual cell. (B) Dot plot showing the percentage of cells expressing the indicated subcluster marker genes. The size of the dot corresponds to the proportion of cells detected and the intensity of the colour, to the expression level (mean log_10_ *p*‐value). (C) Distribution of the four types of immune cells between the left atrial appendage (LAA) and right atrial appendage (RAA). (D) Violin plot of the expression of *LYVE1*, *MRC1* and *CCR2* in macrophages from the LAA and RAA. (E) Heat map of differentially expressed genes (DEGs) in macrophages of the LAA and RAA. (F) Distribution of seven macrophage subclusters between the LAA and RAA. (G) Heat map of DEGs in seven macrophage subclusters. (H) Violin plots showing the expression of representative marker genes in each subcluster. Boxes indicate the lower and upper quartiles (from left to right), the line within each box indicates the median value, and the whiskers show 1.5× the interquartile range (IQR). (I) Distribution of M1, M2 and fibroblast (FB)‐like macrophages between LAA and RAA. For all violin plots above, the centre line shows the median value, the box limits show the upper and lower quartiles, and the whiskers show 1.5× the IQR.

Analysis of 7449 single cells associated with immune cell populations in our unsupervised model indicated that most of them (macrophages, monocytes and lymphocytes) were strongly related, whereas B cells were separated (Figure 4A). Clusters of macrophages, monocytes, lymphocytes and B cells showed heterogeneous distribution on the *t*‐SNE plot and were identified according to the expression of specific markers (Figure 4B). More monocytes, lymphocytes and B cells were present in the RAA group, whereas more macrophages were present in the LAA group (Figure 4C). Notably, macrophages in both the LAA and RAA were resident, as evidenced by the low expression of *CCR2*, a marker of bone marrow‐derived macrophages^38^; however, other resident macrophage markers, *LYVE1* and *MRC1*, were highly and widely expressed, primarily representing CCR2− LYEVE1+MRC1+ macrophages in our tissue samples (Figure 4D). Meanwhile, distinct expression patterns were observed in macrophages between the LAA and RAA. RAA macrophages had higher levels of genes encoding metallothionein (*MT1G*, *M1TX*), which inhibit the production of proinflammatory cytokines^39^ (Figure 4E). After merging the subclusters with similar gene expression profiles, we identified three types of macrophages from seven subclusters (Figure 4F). The FB‐like macrophages of cluster 7 expressed several FB genes, including *DCN* and *FABP5*, and SMC genes, including *COL1A1* and *COL1A2* (Figure 4G). Subclusters 1 and 6 exhibited strong expression of the M1 markers *ILB* and *CD86*, suggesting their composition of M1‐like macrophages. On the other hand, M2 markers *MERTK*, *MRCI* and *STAB1* were expressed in subclusters 2–5 indicating their composition of M2‐like macrophages (Figure 4H).^40^ Proportions of FB‐ and M1‐like macrophages were higher in the LAA than in the RAA (Figure 4I), suggesting that the macrophages underwent phenotypic conversion by responding to tissue remodelling during chronic inflammation in AF.

### 3.6 TFPI, TFPI2 and ADAMTS1 in the AF model

**FIGURE 5:**
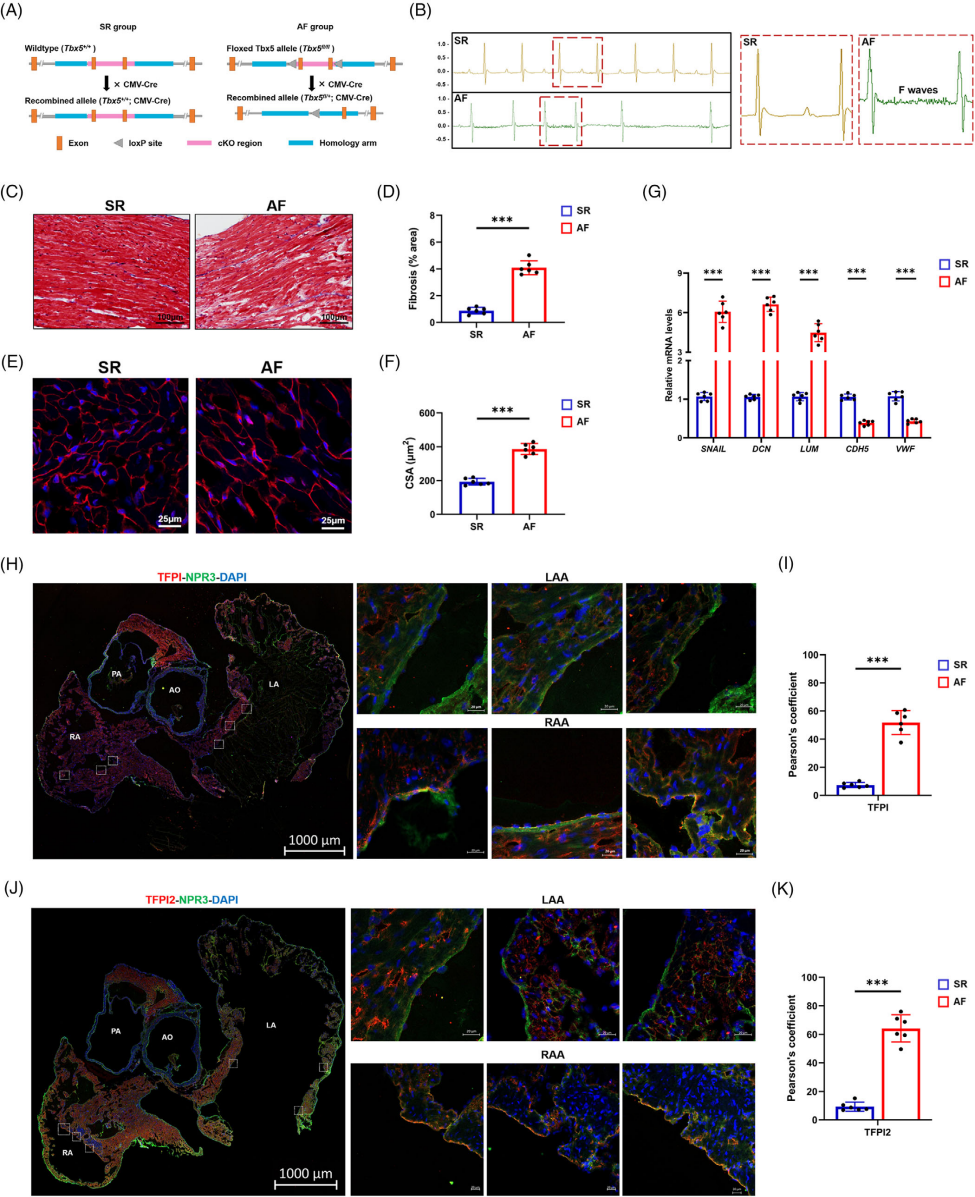
Establishment of the model of atrial fibrillation (AF) mice and expression of tissue factor pathway inhibitor (TFPI) and tissue factor pathway inhibitor 2 (TFPI2). (A) Experimental schema of the model of AF mice. Male CMV‐Cre mice and *Tbx5* ^+/−^ mice (*Tbx5^fl/+^*; CMV‐Cre) were used as sinus rhythm (SR) and AF mice, respectively. (B) Representative electrocardiogram (ECG) of SR mice and AF mice (left), and regular fibrillatory (F) waves indicating atrial flutter are zoomed‐in on the right. (C) Histological images of hearts by Masson trichrome staining (scale bar = 100 μm). (D) Relative fibrotic area in sections as shown in (C). (E) Representative images stained with wheat germ agglutinin (WGA) (red) and DAPI (blue). Scale bar = 20 μm. (F) Quantification of the cross‐sectional area (CSA) of atrial cardiomyocytes shown in (D). (G) Expression of select genes related to endothelial to mesenchymal transition (EndMT) in tissues of the atrial appendages of SR and AF mice. (H) Immunostaining for the TFPI (red) and endocardial endothelial cell (EEC) marker natriuretic peptide receptor 3 (NPR3) (green) in clarified heart sections of AF mice (scale bar = 1000 μm) and zoomed‐in details (small squares, scale bar = 20 μm). (I) Pearson overlap coefficient analysis indicated decreased colocalization of TFPI with NPR3 in left atrial appendage (LAA) and right atrial appendage (RAA). (J) Immunostaining for the TFPI2 (red) and EEC marker NPR3 (green) in clarified heart sections of AF mice (scale bar = 1000 μm) and zoomed‐in details (small squares, scale bar = 20 μm). (K) Pearson overlap coefficient analysis indicated decreased colocalization of TFPI2 with NPR3 in LAA and RAA. Data were expressed as mean ±eSEM (*n* = 6). ***p < .001. LV, left ventricle; LA, left atrium; RA, right atrium; AO, aorta; AV, aortic valve.

As EECs in the LAA of patients with AF had a lower expression of *TFPI* and *TFPI2*, but a higher expression of *ADAMTS1* (Figure 2D,E), we further explored the role of these genes in AF using a mouse model. Floxed mice with the conditional deletion of T‐box transcription factor 5 (*Tbx5^fl/fl^*) were crossed with CMV‐Cre mice to establish the AF mouse model (*Tbx5^fl/+^*; CMV‐Cre) (Figure 5A).^41^ The mice developed spontaneous AF as evidenced by irregular heartbeat on the electrocardiogram (Figure 5B). Along with abnormalities in the cardiac conduction system, we observed significantly higher cardiac fibrosis (Figure 5C,D) and enlarged cardiomyocyte cross sectional area in AF mice than in SR mice (Figure 5E,F). To gain insight into the possible endocardial origin of FBs, we performed RNA analysis on fresh tissues from SR or AF mice. The EndMT marker *SNAIL* and the mesenchymal marker *DCN* were significantly up‐regulated in the AF tissue compared to the SR tissue, whereas the endothelial marker *CDH5* was down‐regulated (Figure 5G), suggesting that FBs within AF tissue were derived from the endocardium by EndMT. Although increased gene expression did not automatically indicate increased protein production, possible cell markers (VWF for ECs, DCN for FBs and NPPA for cardiomyocytes) and selective proteins (TFPI, TFPI2 and ADAMTS1) were confirmed by immunohistochemistry. Brown dots were more concentrated in the endocardium than in other parts, indicating increased expression of TFPI and TFPI2 (Figure S12), which were further investigated using indirect immunofluorescence and NPR3. In the endocardium of a heart with AF, both TFPI (Figure 5H,I) and TFPI2 (Figure 5J,K) were expressed higher in the RAA than in the LAA.

**FIGURE 6:**
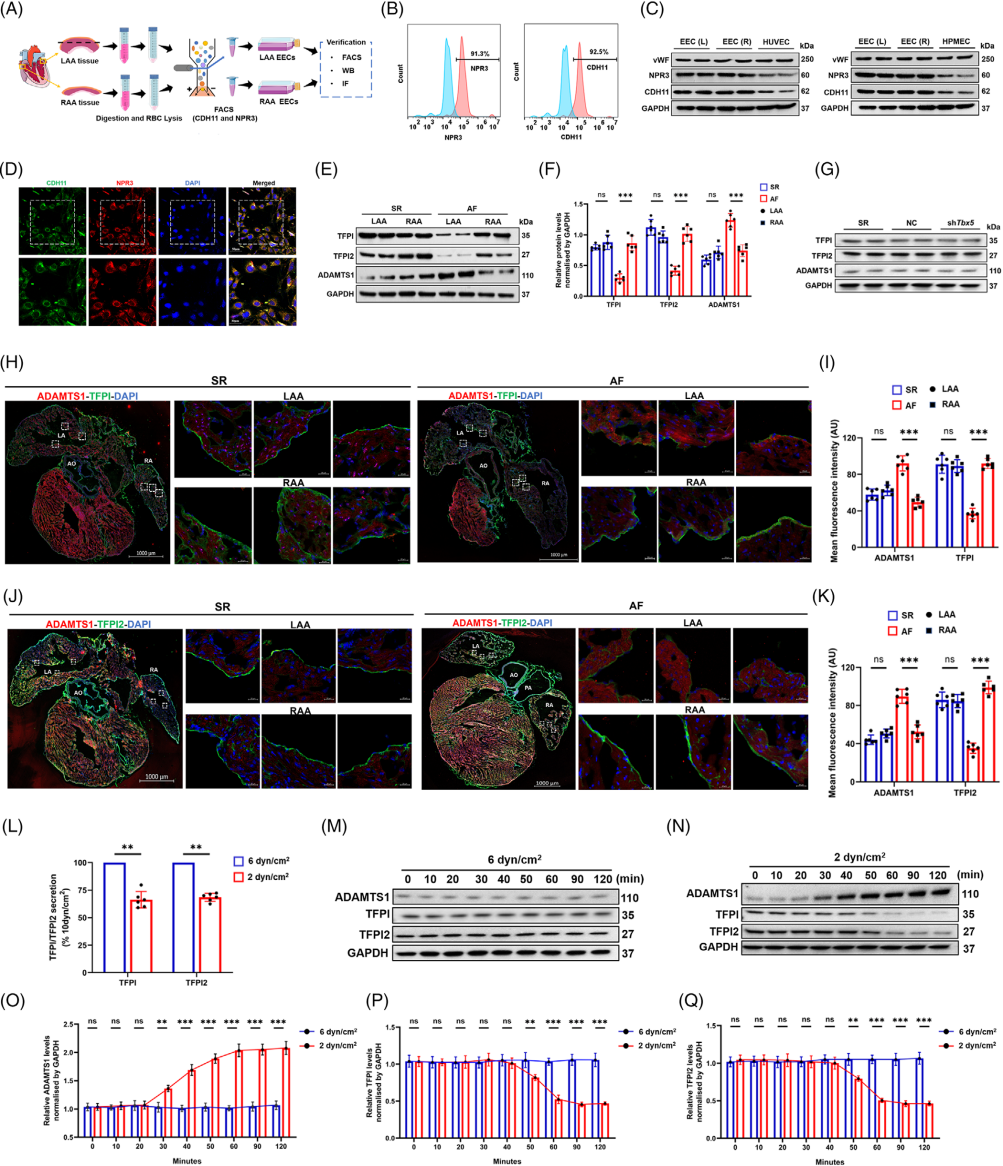
Isolation and localization of endocardial endothelial cells (EECs) for the expression of tissue factor pathway inhibitor (TFPI), tissue factor pathway inhibitor 2 (TFPI2) and a disintegrin and metalloproteinase with thrombospondin motifs 1 (ADAMTS1) in atrial fibrillation (AF) and sinus rhythm (SR) mice. (A) Diagram of digestion, sorting and subsequent verification of EECs isolated from the left atrial appendage (LAA) and right atrial appendage (RAA). (B) FACS data verifying the isolation of EECs with natriuretic peptide receptor 3 (NPR3) and cadherin 11 (CDH11). (C) Western blots (WB) data verifying increased expression of NPR3 and CDH11 in EECs compared to human umbilical vein endothelial cells (HUVECs) (up) and human pulmonary microvascular endothelial cells (HPMECs) (down). (D) Immunostaining data verifying the colocalization of CDH11 (green) and NPR3 (red) in the EECs. (E) WB data showed the expression of TFPI, TFPI2 and ADAMTS1 in the LAA and RAA of AF and SR mice. (F) Quantitative analysis verified that TFPI and TFPI2 decreased, but ADAMTS1 increased in AF of the LAA compared to that of the RAA. (G) TFPI, TFPI2 and ADAMTS1 expression did not change significantly in *Tbx5* knockdown cells. (H) Immunostaining for the localization of ADAMTS1 (red) and TFPI (green) in clarified heart sections (scale bar = 1000 μm) and zoomed‐in details (small squares, scale bar = 20 μm) of the LAA and RAA, respectively. (I) The mean fluorescence intensities of ADAMTS1 and TFPI were calculated; ADAMTS1 is higher but TFPI is lower in LAA. (J) Immunostaining for the localization of ADAMTS1 (red) and TFPI2 (green) in clarified heart sections (scale bar = 1000 μm) and zoomed‐in details (small squares, scale bar = 20 μm) of the LAA and RAA, respectively. (K) Mean fluorescence intensities of ADAMTS1 and TFPI2 were calculated; ADAMTS1 is higher but TFPI2 is lower in LAA. (L) Secretion of TFPI and TFPI2 by EECs in medium with 6 or 2 dyn/cm^2^ shear force exposure. Data were expressed as mean ±eSEM (*n* = 5). (M) EECs in medium were exposed to a shear stress of 6 dyn/cm^2^ against low shear stress, and the expression of ADAMTS1, TFPI and TFPI2 was analysed in time series. (N) EECs in medium were exposed to a shear stress of 2 dyn/cm^2^ against low shear stress, and whole cell lysates were analysed for the expression of ADAMTS1, TFPI and TFPI2 by WB in a time series. (O–Q) The expression of ADAMTS1, TFPI and TFPI2 in (M) and (N) was quantitated after exposure to the indicated shear force. **p < .01. LV, left ventricle; LA, left atrium; RA, right atrium; AO, aorta; AV, aortic valve.

Using FACS based on NPR3 and CDH11 expression, we isolated EECs from LAAs and RAAs of SR and AF mice (Figure 6A,B). Western blot (Figure 6C) and immunocytochemistry (Figure 6D) confirmed higher levels of NPR3 and CDH11 in isolated EECs compared to HUVECs and HPMECs. Next, we verified the basic findings of the scRNA‐seq in patients with AF by quantifying the expression of TFPI, TFPI2 and ADAMTS1 in the EECs of SR and AF mice. The expression of these proteins was not significantly different by cardiac chamber in SR mice. However, in AF mice, the expression of TFPI and TFPI2 was considerably lower, and that of ADAMTS1 was higher in LAA EECs than in those of the RAA (Figure 6E,F), consistent with the results of the transcriptomic analysis of human samples. The extent of the observed differences was unclear due to the presence of AF and/or the lack of *Tbx5* expression. Therefore, we further investigated the effects of *Tbx5* through shRNA transfection targeting *Tbx5* in EECs. The results showed that the expression of TBX5 in EECs treated with sh*Tbx5* was significantly knocked down (Figure S13). ADAMTS1 expression did not change significantly when Tbx5 was knocked down and no significant changes were observed in TFPI or TFPI2 after silencing *Tbx5* (Figure 6G). Furthermore, we examined the fluorescence intensity of these proteins. We observed basal levels of ADAMTS1 and TFPI, with no significant differences between LAAs and RAAs in SR mice (Figure 6H,I). In AF mice, TFPI fluorescence was markedly decreased and ADAMTS1 fluorescence increased in LAAs. Similar changes were also observed when comparing the fluorescence intensity of TFPI2 in LAAs and RAAs of AF mice, which were not visible in SR mice (Figure 6J,K). These experiments indicated that a specific mechanism regulated the expression of these proteins, regardless of the influence of Tbx5.

### 3.7 Effect of shear stress on ADAMTS1, TFPI and TFPI2

Blood stasis and turbulence are considered important factors of thrombogenesis in the LAA during AF, which is characterized by a decrease in wall shear stress in the endothelium.^42^ To determine whether blood flow‐mediated decrease in shear stress was associated with changes in TFPI and TFPI2 expression levels in LAA EECs, we subjected cultured EECs to shear stress at the levels (2 dyn/cm^2^) observed in patients with AF.^43^ The expression of ADAMTS1 expression was up‐regulated, whereas that of TFPI and TFPI2 was down‐regulated by a shear stress of 2 dyn/cm^2^ compared to a stress of 6 dyn/cm^2^ (Figure 6K,L). Regarding anti‐FXa activity, loss of TFPI and TFPI2 activity was also represented by decreased shear stress (Figure 6L). These results suggested that shear stress could induce the expression of ADAMTS1 and reduce the expression of TFPI and TFPI2 in the EECs. To explore the temporal correlation of shear stress with the above proteins, we detected ADAMTS1, TFPI and TFPI2 levels in EECs treated with Flexcell at different time intervals. When treated with 6 dyn/cm^2^, all proteins exhibited expressions similar to their corresponding controls (Figure 6M–Q). However, we recorded a time‐dependent up‐regulation of ADAMTS1 after 30 min with 2 dyn/cm^2^ treatment, reaching a plateau phase at 50 min, whereas the subsequent decrease in TFPI and TFPI2 expression appeared only after 50–90 min (Figure 6M–Q). Notably, TFPI and TFPI2 down‐regulation occurred approximately 20 min after the change in ADAMTS1 expression, which supports that the shear stress‐induced decrease in TFPI and TFPI2 may be dependent on ADAMTS1 in EECs. These data suggested that the expression of ADAMTS1, TFPI and TFPI2 in the EECs was regulated by shear stress, and that ADAMTS1 was associated with TFPI and TFPI2.

### 3.8 ADAMTS1 interacts directly with TFPI and TFPI2

**FIGURE 7:**
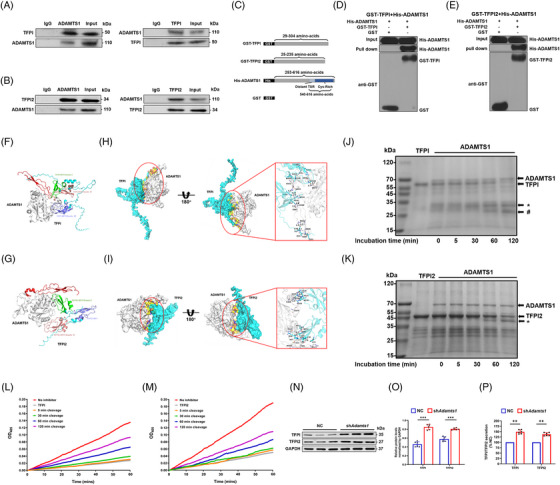
A disintegrin and metalloproteinase with thrombospondin motifs 1 (ADAMTS1) cleaves both tissue factor pathway inhibitor (TFPI) and tissue factor pathway inhibitor 2 (TFPI2) through direct interaction. (A) Interaction between ADAMTS1 and TFPI in endocardial endothelial cells (EECs) of mice by Co‐IP. Mice EECs were subjected to immunoprecipitation (IP) with anti‐TFPI, anti‐ADAMTS1 or anti‐IgG antibodies, followed by western blots (WB) with anti‐TFPI or anti‐ADAMTS1 antibodies. (B) Interaction between ADAMTS1 and TFPI2 in mice EECs by Co‐IP. Mice EECs were subjected to IP with anti‐TFPI2, anti‐ADAMTS1 or anti‐IgG antibodies, followed by WB with anti‐TFPI2 or anti‐ADAMTS1 antibodies. (C) Schematic representation of the fusion proteins used to perform pull‐down and cleavage assays. (D and E) Interaction of ADAMTS1 with TFPI (D) or TFPI2 (E) in vitro by glutathione *S*‐transferase (GST) pull‐down assays. Blots were evaluated using anti‐His or anti‐GST antibodies, and visible His‐ADAMTS1 bands were detected in pull‐down lanes. Input: whole lysate. (F and G) Schematic visualization of the complexes ADAMTS1‐TFPI (F) and ADAMTS1‐TFPI2 (G). Three‐dimensional structure showing ADAMTS1 (grey) and the TFPI domains (F), including Kunitz I (green), Kunitz II (pink) and Kunitz III (purple), or TFPI2 (G), including Kunitz I (green), Kunitz II (pink) and BPT (purple). (H and I) Selected complex examples of ADAMTS1‐TFPI (H) and ADAMTS1‐TFPI2 (I). Grey ribbons represent ADAMTS1, whereas blue ribbons represent TFPI (H) and TFPI2 (I). A view rotated 180° around the *x*‐axis is shown with the enlargement of the interacting surface in a red solid box (right). The red and blue spheres represent oxygen and hydrogen atoms, respectively. The interface surface between ADAMTS1 and TFPI (H) and ADAMTS1 and TFPI2 (I) is coloured yellow, and hydrogen bonds are highlighted in black dashed lines. (J) Time course of purified recombinant ADAMTS1 cleavage from purified recombinant TFPI in vitro at 37°C and analysed by Coomassie Blue staining. Full‐length TFPI (56 kDa) and cleavage fragments (32 kDa* and 27 kDa#) are indicated by arrows. (K) Time course of purified recombinant ADAMTS1 (1 μg) cleavage from purified recombinant TFPI2 (1 μg) in vitro at 37°C and analysed by Coomassie Blue staining. Full‐length TFPI2 (49 kDa) and cleavage fragments (42 kDa*) are indicated by arrows. (L) Purified recombinant TFPI2 was incubated in the presence or absence of purified plasma ADAMTS1 for various incubation times, and subsequent FX binding was assessed in an enzyme linked immunosorbent assay (ELISA) setup for 180 min. (M) Purified TFPI2 was incubated in the presence or absence of purified plasma ADAMTS1 for various incubation times, and subsequent FX binding was assessed in an ELISA setup for 180 min. (N) WB analysis of EECs transfected with sh*Adamts1* RNA or negative control (NC) vector. (O) Quantification of TFPI and TFPI2 after transfection with different plasmids. Data were expressed as mean ± SEM (*n* = 6). ***p < .001. (P) Secretion of TFPI and TFPI2 by EECs in a medium transfected with sh*Adamts1* or NC. Data were expressed as mean ±eSEM (*n* = 6). **p < .01. IB, immunoblotting; Cys, cysteine.

The opposite changes in the expression of ADAMTS1 and TFPI/TFPI2 in response to shear stress suggested a potential link between these proteins. To explore whether ADAMTS1 could interact with TFPI and/or TFPI2, we first performed a Co‐IP on EEC extracts for TFPI and TFPI2. Anti‐ADAMTS1 antibodies but not control IgG could immunoprecipitate ADAMTS1, TFPI (Figure 7A) and TFPI2 (Figure 7B). Expectedly, ADAMTS1 in EECs was also immunoprecipitated using an antibody against TFPI (Figure 7A) or TFPI2 (Figure 7B). These data indicated that endogenous TFPI and TFPI2 interacted with ADAMTS1 in vivo. Subsequently, truncated fragments of TFPI and TFPI2 were constructed for further pull‐down assay to test whether the association between ADAMTS1 and TFPI or TFPI2 was based on their direct interaction (Figure 7C). His‐ADAMTS1 was specifically precipitated by glutathione S‐transferase (GST)‐TFPI (Figure 7D) or GST‐TFPI2 (Figure 7E) but not by GST protein alone, confirming the direct interaction of ADAMTS1 with TFPI and TFPI2 in vitro. Currently, no structural information is available for full‐length TFPI, TFPI2 or ADAMTS1. Hence, we applied the corresponding aa sequence (Figure 7C) used in pull‐down assays along with the existing information of identical aa sequences with known structures to construct structural models with AlphaFold2. Sequence analysis showed that both TFPI and TFPI2 contain an acidic N‐terminal region, followed by three repeated Kunitz (K)‐type domains (K1, K2 and K3) and a highly basic C‐terminal domain (Figure S14). ZDOCK, a high‐throughput strategy that predicts protein binding affinity well, was used to predict the potential binding regions of TFPI and TFPI2. The best scoring complex model was further analysed for binding patterns. As indicated in Figure 7F, three‐dimensional simulations revealed that TFPI could bind to ADAMTS1 in two domains: K1 and K2 (energy score: 1132.93). For TFPI2, the docking was guided by two key observations, K1 and K3, which may be involved in the interaction (energy score: 1148.00; Figure 7G). The binding domain of ADAMTS1 at the interface of the best scoring complex is the 540−616 aa sequence, which contains a distant TSR domain and a Cys‐rich area (Figure 7H,I). Detailed information on the interaction forces is shown in Tables S3 and S4, which were mainly defined as strong interactions derived by the hydrogen bond. The structural similarities between TFPI and TFPI2 prompted us to assume that shared domains are more likely to be involved in the interaction process. All the above indicated that TFPI and TPI2 could directly interact with ADAMTS1, and the distant TSR domain of ADAMTS1 is the potential target for the K1 domain in both TFPI and TFPI2.

### 3.9 ADAMTS1 cleaves TFPI and TFPI2 and inhibits Anti‐FXa activity

ADAMTS1 is a metalloproteinase that cleaves ECM proteins such as aggrecan^44^ and versican^45^; however, several novel substrates, including TFPI2 and thrombospondins 1 and 2, have recently been identified in vitro.^46^, ^47^ Therefore, we investigated whether ADAMTS1 could cleave TFPI and/or TFPI2 by incubating purified recombinant His‐ADAMTS1 with purified recombinant GST‐TFPI or GST‐TFPI2. The results indicated that ADAMTS1 could cleave TFPI in a time‐dependent manner, as evidenced by the gradual disappearance of full‐length TFPI and the appearance of cleavage fragments (Figure 7J). ADAMTS1 also readily cleaved TFPI2, generating additional products of approximately 42 kDa (Figure 7K). The loss of MW of approximately 6 kDa was consistent with the observation of Rodríguez.^48^ To assess whether ADAMTS1 cleavage affected the functional status of TFPI and TFPI2, we investigated their anti‐FXa/VIII activities using ELISA. Figure 7L shows that anticoagulant activity of TFPI progressively decreased after treatment with ADAMTS1. A time‐dependent decrease in antifactor VIII activity was also observed for TFPI2 incubated with ADAMTS1 (Figure 7M). In general, these results indicated that ADAMTS1 could inactivate TFPI and TFPI2 by proteolytic degradation. Finally, to verify the specificity of the proteolytic effect of ADAMTS1 on TFPI and TFPI2, ADAMTS1 was silenced in EECs in vitro using *Adamts1*‐specific shRNA, which was confirmed by western blotting (Figure S15). Knockdown of *Adamts1* expression in EECs resulted in increased levels of full‐length TFPI and TFPI2 (Figure 7N,O) in the cell supernatant and increased anti‐FXa activity compared to the negative control (Figure 7P). These results indicated that ADAMTS1 could directly interact with and proteolytically degrade TFPI and TFPI2, thus blocking their anti‐FXa activity, which can result in the local imbalance of the coagulation cascade and promote thrombosis in the atrium.

## 4 DISCUSSION

**FIGURE 8:**
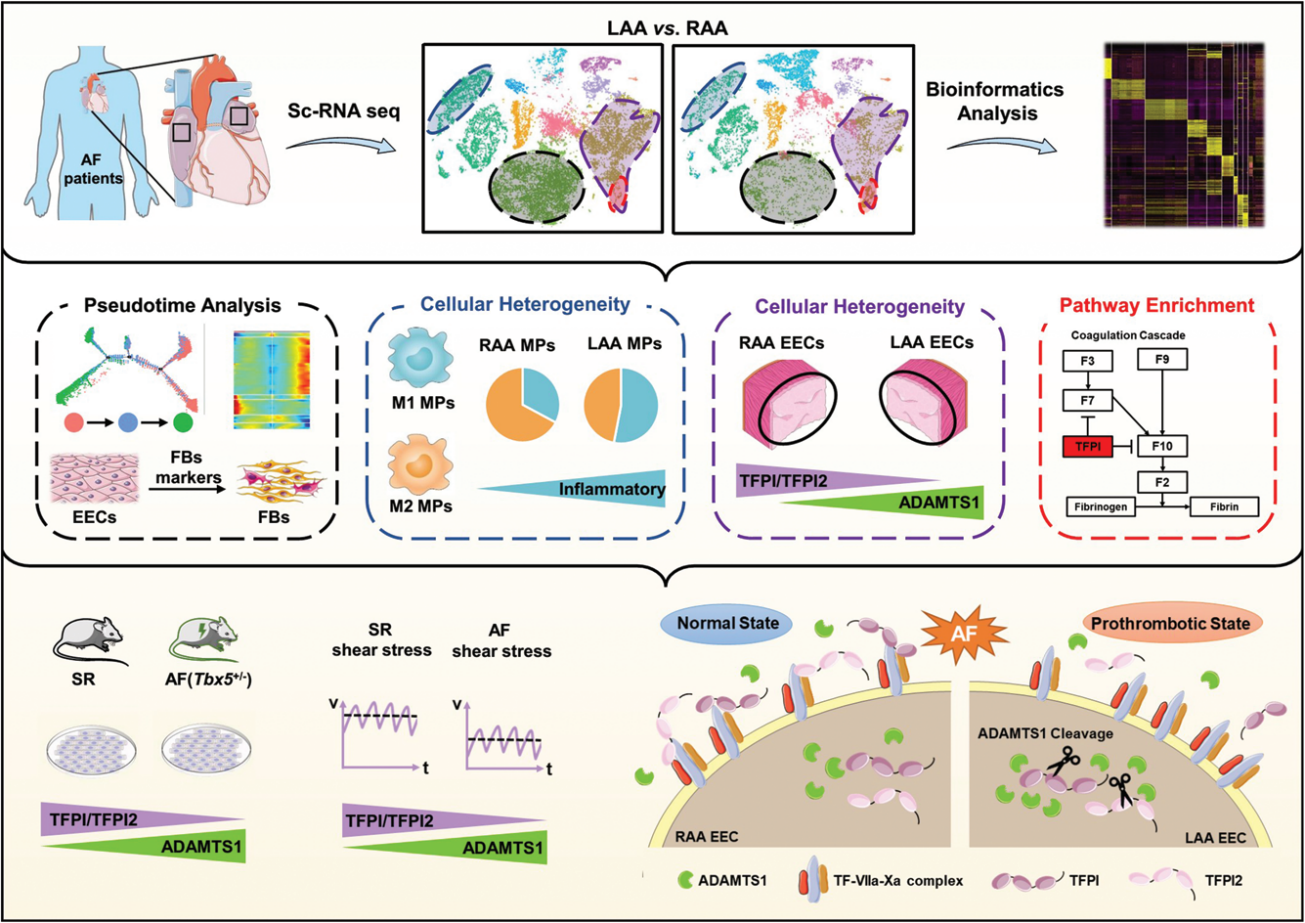
Graphical abstract. We conducted a single cell transcriptomic analysis to determine chamber‐specific characteristics of the atrial appendages. Potential mechanisms of pathological changes related to atrial fibrillation (AF), including atrial fibrosis through endothelial to mesenchymal transition (EndMT), macrophage polarization and imbalance of local anticoagulation in endocardial endothelial cells (EECs). Disturbed flow during AF‐reprogrammed EECs triggers high a disintegrin and metalloproteinase with thrombospondin motifs 1 (ADAMTS1) expression and decreased tissue factor pathway inhibitor (TFPI) and tissue factor pathway inhibitor 2 (TFPI2), resulting in the prothrombotic state of left atrial appendage (LAA).

In our study, we performed, at a single‐cell level, a comparative analysis of the transcriptional landscapes specific to LAAs and RAAs affected by AF. First, we characterized the genes and functional pathways in 32 326 cells (1749 genes per cell). Second, we revealed the differences in LAA and RAA transcriptional programmes across the major cell types and subtypes, which revealed the roles of complex cell ensembles in the fibrosis, inflammation and endocardial dysfunction observed in AF. Third, we verified the differential functional activity of EECs in LAAs and RAAs of AF mouse models, which confirmed the low expression of the anticoagulant factors TFPI and TFPI2 and high expression of ADAMTS1 in LAA EECs. Finally, we found that the down‐regulation of local levels of TFPI and TFPI2 could be attributed to ADAMTS1‐mediated proteolytic degradation, leading to the imbalance of coagulant–anticoagulant reactions in the LAA. Therefore, we disclosed a previously unknown mechanism potentially involved in the susceptibility of the LAA to thrombogenesis during AF, which should contribute to our understanding of the pathology of AF (Figure 8).

Increasing evidence points to the role of specific cell populations in heart health and disease^25^, ^26^, ^49^, ^50^; however, little is known about the pathogenesis underlying AF development because the data in human samples are limited. Our study supplemented these data by analysing paired atrial appendages of patients with AF, which are the sites where thrombus formation mainly occurs. In both LAAs and RAAs, we identified the same cardiac cell types as those previously reported; however, most of them exhibited site‐specific differences in terms of abundance and expression profiles, which is consistent with the view that AF is an LA‐related disease.^51^ Notably, the EC population exhibited unexpected heterogeneity in subtype composition, indicating that ECs may undergo profound transcriptional and functional changes during AF. In healthy adult hearts, ECs consist of two subpopulations that express FB or cardiomyocyte genes.^26^ Our results suggest the existence of an even more heterogeneous EC population, which can be due to the chronic pathological processes associated with AF. However, we did not identify subsets of coronary artery ECs (FABP4/CD36 positive) and epicardial cells (TBX18 positive), partly because the atrial auricular tissues were analysed without the epicardium. Our results indicate that the LAA contained fewer EECs but more FBs, suggesting that AF may accelerate the transformation of EECs into mesenchymal cells and FBs and also promote their dysfunction and apoptosis. The EECs of the LAA were characterized by a decreased expression of TFPI and TFPI2 but an increased expression of thrombomodulin and FVIII, suggesting the dysregulation of the blood coagulation cascade. Collectively, these effects can make the LAA more prone to thrombosis than the RAA. Furthermore, a recent HEART‐CLOT study in patients with permanent AF showed that the LAA presents a local prothrombotic state, as reflected by reduced clot permeability, making it susceptible to forming compact fibrin clots compared to the other heart chambers.^52^

Compared to the RAA, the LAA exhibited fewer EECs, more FBs and more severe fibrosis. The results also support the activated phenotype concept as indicated by the higher proportion of CILP‐positive cells in the LAA than in the RAA, which can be instrumental in remodelling the extracellular environment through increased deposition and rearrangement of the ECM,^30^ consistent with severe fibrosis observed in the LAA. Activated FBs and the associated excessive production of ECM can cause cardiomyocyte isolation, thus interfering with electrical continuity and stimulating the progression of AF to chronic forms. The maturation of collagen and the production of ECM proteins, namely fibronectin, tenascin C and thrombospondin 1, are considered the main factors that promote atrial fibrosis.^53^ GO analysis also indicated that platelet degranulation could be another process that contributes to fibrosis and electrophysiological and structural remodelling. Furthermore, increased expression of profibrotic genes was observed in many non‐FB cells (viz. FB‐like ECs), suggesting that atrial fibrosis induced by AF could be the result of the activity of several cell types or from cells in a transition state. Thus, the pseudotime analysis, including the unknown cell population, showed a continuous transformation from ECs, especially EECs and FB‐like ECs, to unknown cells and then to FBs, indicating that the unknown cell group represented an intermediate state in the transformation of phenotypic cells during AF development. These results explain the origin of the increase in FB population in the LAA and offer a detailed profile of cell groups that could initiate the EndMT process involved in cardiac fibrosis.

EECs cover the inner surface of the atrium and produce various proteins,^54^ including TFPI, which neutralizes the FVIIa‐TF complexes, FVIIa and FXa to inhibit the exogenous coagulation pathway.^55^ Several observational studies have indicated that the surgical treatment of LAAs, either by percutaneous LAA occlusion or surgical exclusion procedure, is associated with persistent changes in coagulation factor activity^56^ and the regulation of heart metabolism.^57^, ^58^ As an alternatively spliced anticoagulant protein, a proportion of TFPI molecules are truncated to a variable extent at the COOH‐terminal end, which compromises inhibitory activity. Inconsistent changes in TFPI levels in patients with thromboembolic diseases^59^ and increased circulating TFPI levels in patients with acute myocardial infarction are attributed to the destruction of the ECs and the extensive release of TFPI into the blood.^60^ In contrast, patients with chronic heart disease have lower plasma levels of TFPI due to the long‐term dysfunction of ECs and decreased TFPI synthesis.^61^ These contradictory results point to the association between the function of ECs and the degree of inflammation in the development of heart disease. In this study, all patients had persistent AF and no other inflammatory diseases; therefore, the analysis of their tissue samples should reveal the relationship between dysfunctional anticoagulant synthesis in ECs and thrombosis in the LAA and RAA. AF itself is known to enhance platelet aggregation and coagulation by increasing the levels of beta‐thromboglobulin and platelet factor 4.^62^ Meanwhile, decreased *TFPI* mRNA levels in the atrial endocardium in a rapid atrial pace rat modelled to an acute deficiency of endocardial protection against clotting.^63^ This endocardial dysfunction was significantly reversed by the down‐regulation of antithrombotic molecules, including eNOS, TFPI and TM using the AT1 receptor blocker.^64^ However, the aforementioned data were obtained for both atria without distinction between the left and right chambers. We focused on the endocardium of LAAs and RAAs, where most intracardiac thrombi are typically localized, specifically targeting DEGs in EECs, and revealed down‐regulation of TFPI in LAA EECs, which was confirmed in the AF mouse model. TFPI2 is another anticoagulant decreased in EECs from LAAs of AF patients. In addition to its anticoagulation effects, TFPI2 inhibits the proliferation and migration of atrial FBs and induces their apoptosis.^65^ Therefore, the decrease in TFPI2 could be associated with the higher proportion of FBs observed in the LAA. Therefore, the inability of EECs to produce sufficient levels of TFPI or TFPI2, and the decrease in their population in the LAA, could contribute to the preferential formation of thrombi in the LAA rather than in the RAA of patients with persistent AF.

Another important factor for decreased TFPI and TFPI2 activity was their proteolytic degradation by ADAMTS1,^48^ an extracellular protease of the ADAMTS family. The modular structure of ADAMTS1 allows it to exercise catalytic activity via a multiplicity of protein–protein interactions in the presence of a disintegrin domain. Here, ADAMTS1 colocalized with TFPI in the endothelium, and the interaction of ADAMTS1 with TFPI and TFPI2 in EECs was confirmed by coimmunoprecipitation and pull‐down assays. The degradation products of TFPI and TFPI2 generated by ADAMTS1 were similar to those produced by MMP7 and MMP9,^66^ indicating a similarity at the cleavage sites. Tension and stress stimulations are capable of regulating ADAMTS1 to remodel the ECM and promote angiogenesis. Irregular contraction and LA wall rigidity in AF prevented effective blood flow to the ventricles, resulting in blood stasis and decreased shear strain rate in the LAA. Studies have identified the exact shear stress in the LA of patients with AF, a mean of 2−3 dyn/cm^2^.^42^, ^43^ Therefore, we simulated shear stress to incubate EECs and detect the increased expression of ADAMTS1 and decreased activity of TFPI and TFPI2. Therefore, abnormal haemodynamics‐induced reduction of shear stress in AF patients can further alter ADAMTS1 levels in ECCs. Accumulated ADAMTS1 cleaved TFPI and TFPI2 results in decreased release of complete forms of TFPI and TFPI2 and promotes thrombin generation due to the insufficient inhibition of the FVIIa‐TF complex and FXa. However, the corresponding molecular mechanism of how changes in shear stress impact ADAMTS1 transcription and change the EEC phenotypes requires further investigation.

Our study has a few limitations. First, there was unintended surgical sample and cell‐specific biases in capture efficiency. Technology for removing background RNA, identifying nuclear doublets, and performing batch correction is imperfect. Despite the correction, droplets are likely to retain background signals. The aforementioned methods could affect the cell‐type composition, especially when observing the expression of specific genes in other cell types. Second, the number of samples in our study was also limited because few patients agreed to provide RAA samples. Although the current study was conducted on limited human samples, subsequent analyses of WB, IF and other analyses were performed to verify preliminary results in AF mice, namely the high level of ADAMTS1 and the low level of TFPI and TFPI2 in LAA EECs. Third, there were no control samples for comparison because it was difficult to acquire tissues from healthy participants. Validating genetic changes and TF modules remains a challenge, especially in heterogeneous samples from patients with AF. Although we selected a public database for healthy controls, the nuclear transcriptomes represented only a part of the total mRNA and differed from the mRNA present in the cytoplasm. Therefore, further studies are required to examine the concordance of the two transcriptomes and clarify their differences. At last, our quality control procedure discarded parts of cardiomyocytes as a result of large count of mitochondrial genes in cardiomyocytes; thus, the number and gene expression in cardiomyocytes were less reliable and accurate than the non‐cardiomyocytes.

## 5 CONCLUSIONS

In conclusion, we report, for the first time, the single‐cell landscape of the human fibrillating atrium, which should improve understanding of the pathology of AF. Our results uncover the mechanism that underlies the susceptibility of the LAA to thrombus formation in patients with AF, which is local coagulation/anticoagulation imbalance due to decreased TFPI/TFPI2 production and increased ADAMTS1 release by EECs. Our results reveal a novel mechanism and will aid in the development of potential antithrombotic and anticoagulative strategies to treat AF.

## CONFLICT OF INTEREST STATEMENT

All authors declare no conflict of interests.

## Supporting information

Supporting Information

## Data Availability

Raw data associated with the main and supplementary figures are available upon reasonable request. Raw fastq.gz files from scRNA‐seq have been deposited to GEO under an access number (Submission ID: SUB11167988 and SUB11200864; BioProject ID: PRJNA815461), which can be retrieved from the following website: http://www.ncbi.nlm.nih.gov/sra/PRJNA815461.
