# Supplementary material for: Single‐cell RNA sequencing reveals a mechanism underlying the susceptibility of the left atrial appendage to intracardiac thrombogenesis during atrial fibrillation
Source: Clin Transl Med. 2023 Jun 5;13(6):e1297. doi: 10.1002/ctm2.1297 (PMC10242273; doi:10.1002/ctm2.1297)
Supplement: Supplementary file 1 — Supporting Information [file CTM2-13-e1297-s001.docx]

**Supplemental Material**

**Single-Cell RNA Sequencing Reveals a Mechanism Underlying the Susceptibility of** **the Left Atrial Appendage to Intracardiac Thrombogenesis During Atrial Fibrillation**

1. **Supplemental Methods**
2. **Supplemental Figures**

**3. Supplemental Tables**

**1 Supplemental Methods**

**1.1 Histological analysis**

Mouse hearts isolated from mice with AF and SR were excised, fixed in 4% paraformaldehyde, dehydrated in graded concentrations of ethanol, immersed in xylene and embedded in paraffin. Subsequently, 5 μm-thick sections were cut on a microtome, stained with hematoxylin-eosin and Masson trichrome stain, and examined using light microscopy (BX63, Olympus). Six mice from each group were subjected to histological analysis. ImageJ software (RRID:SCR_003070) was used to quantify histological endpoints.

**1.2 Isolation and treatment of primary mouse EECs**

The EECs were isolated as previously described [1]. Briefly, each mouse was euthanized with CO_2_ and its chest was carefully opened to remove the heart, which was washed three times with 50 mL of cold phosphate buffered saline (PBS) to remove excess blood. The left and right atria were carefully cut to avoid contamination of their outer surfaces and minced into 1 mm^3^ pieces under a microdissection microscope (LMD6, Leica, Wetzlar, Germany). Atrium tissues (with endocardium) were placed in separate 5 mL tubes containing 1 mL of digestion buffer for 10 min at 37 °C. The reaction was terminated by adding 4 mL of extracellular matrix (ECM) containing 10% FBS, 50 U/mL penicillin / streptomycin, and 1% L-glutamine. The tubes were centrifuged at 300 × *g* for 10 min at room temperature (RT) and the pellets were resuspended in 1–2 mL 1× RBC lysis buffer (00-4333-57, eBioscience). Subsequently, the cells were resuspended in endothelial cell (EC) medium, seeded in cell culture plates, and kept in an incubator of 5% CO_2_ at 37 °C; the medium was changed every two days. *Adamts1* expression in EECs was inhibited using mouse-specific short hairpin RNA (shRNA) (Genepharma). The cells were then transfected with *Adamts1* or an empty control vector using Lipofectamine 3000 (Invitrogen, Waltham, MA, USA) according to the manufacturer's instructions.

**1.3 EECs purification using FACS**

Cell samples containing EEC were centrifuged, washed three times in PBS, stained with a mixture of anti-natriuretic peptide receptor 3 (NPR3) AF647 antibody (1:200; NBP2-72881; Novus, Centennial, CO, USA) and anti-CDH11 AF488 antibody (1:200; FAB17901G, R&D Systems, Minneapolis, MN, USA) for 45 min in the dark at 4 °C, and washed twice and transferred to a cell strainer to obtain single-cell suspensions. Cells were first gated on 4′,6-diamidino-2-phenylin-dole (DAPI) to exclude debris, and then sorted as NPR3-AF647 positive and CDH11-AF488 positive cells in Eppendorf tubes with culture medium using a flow cytometer (Beckman, Brea, CA, USA). The EECs were resuspended in EC medium and incubated in a 5% CO_2_ incubator at 37 ° C for verification.

**1.4 Western blotting**

Total proteins were extracted from isolated EECs, human umbilical vein endothelial cells (HUVECs; BFN607200285; Cell Bank of the Chinese Academy of Sciences, Shanghai, China), and human pulmonary microvascular endothelial cells (HPMECs; BFN60804388; Cell Bank of the Chinese Academy of Sciences) using Radioimmunoprecipitation (RIPA) lysis buffer (Beyotime Biotechnology, Shanghai, China) and analyzed using western blotting. Protein concentration was determined using the Bicinchoninic acid (BCA) assay (P0012; Beyotime Biotechnology). Furthermore, the same protein amounts were subjected to sodium dodecyl sulfate polyacrylamide gel electrophoresis (SDS-PAGE) and transferred to polyvinylidene fluoride membranes (Millipore, Burlington, MA, USA). The membranes were blocked with 5% fat-free milk (D8340; Solarbio, Beijing, China) in Tris buffered saline (AR0031; Boster Bio, Pleasanton, CA, USA) containing 0.5% Tween-20 (T8220; Solarbio). After incubation with primary antibodies against NPR3 (ab97389; Abcam, Cambridge, UK), CDH11 (H00001009; Novus), von Willebrand factor (VWF; ab174290; Abcam), TFPI (ab180619; Abcam), TFPI2 (ab186747; Abcam), ADAMTS1 (H00009510; Novus), and beta-actin (AF5003; Beyotime Biotechnology) at 4 °C overnight, membranes were incubated with secondary antibodies. Protein bands were detected using enhanced chemiluminescence and quantified using Image QuantTL software (GE Healthcare, Buckinghamshire, UK).

**1.5 Immunohistochemistry**

AF and SR mouse hearts were snap frozen in an optimal cutting temperature compound (C2076; Bioss Biotechnology), cut into 4 μm-thick sections, and incubated in blocking buffer for Immunol staining (P0260; Beyotime Biotechnology) for 10 min to block endogenous peroxidase activity. The sections were subjected to a quick antigen extraction solution (P0090; Beyotime Biotechnology), blocked with 5% normal goat serum, and incubated with primary antibodies against VWF (1:200; ab9378; Abcam), DCN (1:200; ab277636; Abcam), natriuretic peptide A (NPPA; 1:200; ab190001; Abcam), TFPI (1:100; ab180619; Abcam), and TFPI2 (1:100; ab186747; Abcam) for 24 h at 4 °C, then with biotinylated secondary antibody (1:100; P0101; Beyotime Biotechnology), and counterstained with hematoxylin (C0107; Beyotime Biotechnology) for 1 min. The slides were cleared three times with xylene, cover slipped with mounting medium, and observed under a microscope (BX63; Olympus, Tokyo, Japan).

**1.6 Immunofluorescence**

Frozen tissue sections or EECs were fixed in 4% paraformaldehyde (P0099; Beyotime Biotechnology) at RT for 10 min, washed three times with PBS, permeabilized with 0.1% Triton 100-X (P0096; Beyotime Biotechnology) at RT for 30 min, washed three times with PBS, and blocked with blocking buffer (P0102; Beyotime Biotechnology) at 37 °C for 30 min. The samples were then incubated with primary antibodies against NPR3 (1:100; ab97389; Abcam), CDH11 (1:500; H00001009; Novus), TFPI (1:100; ab260042; Abcam), TFPI2 (1:500; NBP1-57942; Novus), and ADAMTS1 (1:100; H00009510; Novus) at 4 °C overnight. After washing twice with PBS, samples were stained with fluorescent secondary antibodies, fluorescein isothiocyanate (FITC)-conjugated (1:500; A0562; Beyotime Biotechnology) or Cy3-conjugated (1:100; A0507; Beyotime Biotechnology) at 37 °C for 1 h, and observed under a laser scanning confocal microscope at 100–400 Hz. Samples not incubated with primary antibodies were used as a negative control. The images were analyzed using LAS X software (Leica) and Image-Pro Plus 5.0 (Media Cybernetics, Rockville, MD, USA).

**1.7 Real-time reverse transcription-PCR** (**RT-PCR)**

Total RNA from the right cardiac tissue was extracted from murine using Trizol reagent (Invitrogen) according to the manufacturer's instructions. Real-time (RT)-PCR was performed to investigate the expression of endothelial-mesenchymal transition (EndMT)-related genes. Briefly, the *Snail*, *Dcn*, *Lum*, *Vwf,* and *Cdh5* primer sequences were designed by Shanghai Shenggong Biology Company (Shanghai, China) and dissolved in Trizol reagent (Thermo Fisher Scientific). Total RNA was reverse transcribed into cDNA according to the manufacturer's procedure for the PrimeScript RT Reagent Kit (TaKaRa, Kusatsu, Japan). Finally, a fluorescence quantitative RT-PCR mixture was prepared with SYBR Premix Ex TaqⅡ. Amplification of EndMT-related genes was achieved by PCR. The expression of target genes was normalized to that of glyceraldehyde-3-phosphate dehydrogenase (GAPDH). Data were analyzed using the comparative Ct method, and each experiment was performed in triplicate.

**1.8 Coimmunoprecipitation**

Coimmunoprecipitation was performed as previously described [2] by immunoprecipitating ADAMTS1 with antibodies against TFPI (AF-2974; R&D Systems) or TFPI2 (ab186747; Abcam). Briefly, 800 μg of total protein extracts in RIPA buffer (50 mM Tris-HCl, pH 7.4, 1% NP-40, 0.25% Na-deoxycholate, 150 mM NaCl, and 1 mM EDTA) were pre-cleared with 50 µL anti-rabbit IgG IP beads (eBioscience) and 15 μL Protein-G sepharose beads (GE Healthcare) at 4 ° C for 30 min, centrifuged at 10000 × *g* for 5 min at 4 °C, and the supernatant was incubated with 15 μL Protein-G sepharose beads coated with primary antibody on ice for 1 h with mixing. Immune complexes were collected by centrifugation at 10,000 × *g*, washed three times with RIPA/PBS (1:3), mixed with SDS sample buffer, heated at 100 °C for 10 min, separated in 12% SDS-PAGE minigels, and analyzed using western blotting with anti-ADAMTS1 antibodies (PA5-47790; Invitrogen) and anti-TFPI2 antibodies (ab186747; Abcam). Protein bands were visualized using enhanced chemiluminescence.

**1.9 Glutathione S-transferase (GST) pulldown assays**

*E. coli* BL21 cells expressing GST-tagged TFPI or -TFPI2 or His-tagged ADAMTS1 were lysed in lysis buffer containing 1% Triton X-100 for 30 min on ice, centrifuged at 15000 × *g* for 15 min, and the supernatant was used for GST pulldown assays. Briefly, equal amounts (0.5 mg) of GST-tagged TFPI or TFPI2 were mixed with His-tagged ADAMTS1, incubated on ice for 3 h, and loaded onto glutathione Sepharose 4 B resin columns. After washing five times with wash buffer, proteins were eluted with wash buffer supplemented with 15 mM reduced glutathione, separated in 12% SDS-PAGE gels, transferred to polyvinylidene fluoride membranes, and probed with anti-His and anti-GST antibodies (Sigma-Aldrich, St. Louis, MO, USA; Merck KGaA, Darmstadt, Germany). GST and His (Wuhan Genecreate, Wuhan, China) served as negative controls. Three replicates were used for each pull-down assay.

**1.10 Rigid-Body docking**

To predict the binding affinity of each of TFPI2 and TFPI to ADAMTS1, we used a rigid-body protein–protein docking approach using the coupling software: ZDOCK version 3.0.2 [3, 4]. ZDOCK is a grid-based docking algorithm that uses fast Fourier transforms to accelerate an exhaustive search in the 6D rotational and translational space, sampling the three Euler angles with a 6° or 15° spacing, and the three translational degrees of freedom with a 1.2 Å spacing. For each set of rotational angles, only the best scoring translation is retained, which results in 3600 or 54 000 predictions for 15° or 6° rotational sampling, respectively. The predictions are ranked according to the ZDOCK scoring function, which combines shape complementarity, electrostatics, and desolvation. Here, we used 15° sampling, resulting in 3600 docking decoys per test case. The higher the Z score value, the better the binding affinity for the protein–protein complex. Each docking setting produces the top 100 docking results. The conformation with the best docking energy was selected for structural extraction for subsequent research.

**1.11 Shear stress experiments**

Shear stress was generated in EEC monolayers using a Flexcell fluid shear stress device, as previously described [5]. Briefly, EECs were transferred to a Bioflex 6-well plate (Flexcell), cultured on a dome-shaped loading post until 90% confluence, and subjected to different levels of shear stress for 24 h, while the control samples were placed under a nonstretching plate for the same time. At the end of the experiment, cells were harvested, centrifuged, and analyzed using western blotting.

**1.12 Statistical analysis**

For the analysis of the scRNA-seq data, statistical analyses were performed using the Seurat package in R (v3.5.1) as described above. For the rest of the experiments, a two-tailed unpaired t-test was used to assess significance, and differences among more than two groups were assessed by one-way analysis of variance (ANOVA). The differences were considered statistically significant at P* < 0.05, P** < 0.01, and P*** < 0.001. Graphs were generated using GraphPad Prism (v8.4.0; GraphPad Software, San Diego, CA, USA) using data represented as mean ± SEM. The number of biological replicates per group is described in the figure legends. For all in vivo studies, investigators were blinded from mouse genotypes to ensure unbiased data collection.

1. Klein A, Bayrau B, Miao Y et al. (2020) Isolation of Endocardial and Coronary Endothelial Cells from the Ventricular Free Wall of the Rat Heart. Journal of visualized experiments : JoVE

2. Lin JS, Lai EM (2017) Protein-Protein Interactions: Co-Immunoprecipitation. Methods in molecular biology (Clifton, N.J.) 1615:211-219

3. Chen R, Li L, Weng Z (2003) ZDOCK: an initial-stage protein-docking algorithm. Proteins 52:80-87

4. Mintseris J, Pierce B, Wiehe K et al. (2007) Integrating statistical pair potentials into protein complex prediction. Proteins 69:511-520

5. Matheson LA, Fairbank NJ, Maksym GN et al. (2006) Characterization of the Flexcell Uniflex cyclic strain culture system with U937 macrophage-like cells. Biomaterials 27:226-233

**2 Supplemental Figures**


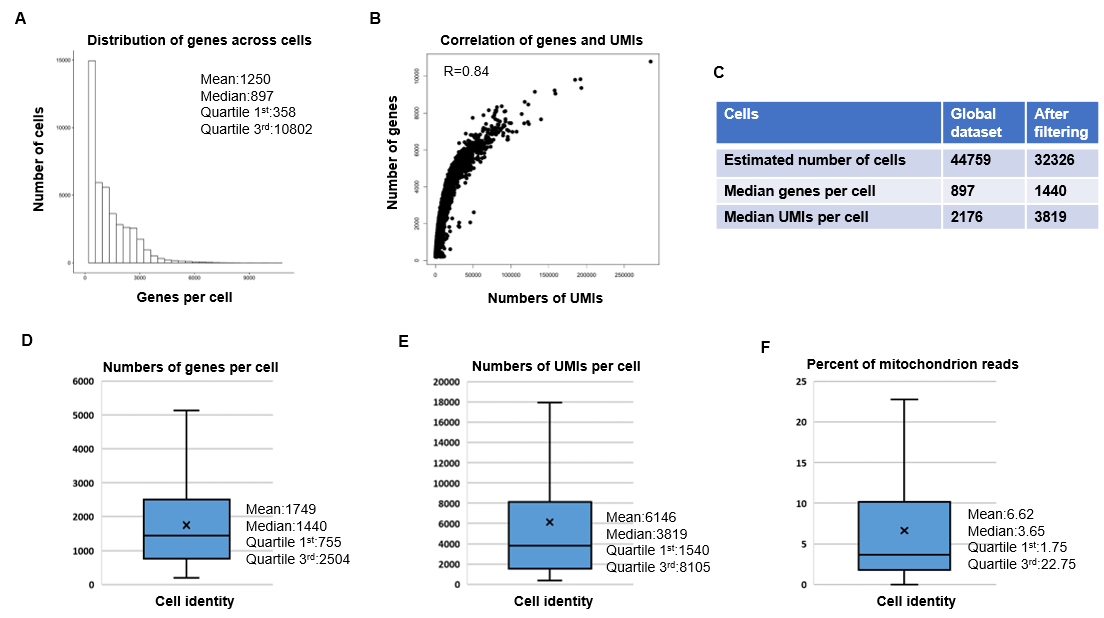


**Figure S1 Viability of cells and data filtering**

(A) Distribution of the total number of unique reads per cell.

(B) Correlation between genes and UMIs in cells.

(C) Summary of quality metrics for single-cell RNA sequencing dataset before and after data filtering.

(D) Box plot indicating the number of genes detected in individual cells included in the analysis.

(E) Box plot indicating the number of UMIs detected in individual cells included in the analysis.

(F) Box plot indicating the percent of mitochondrion reads in cells included in the analysis.


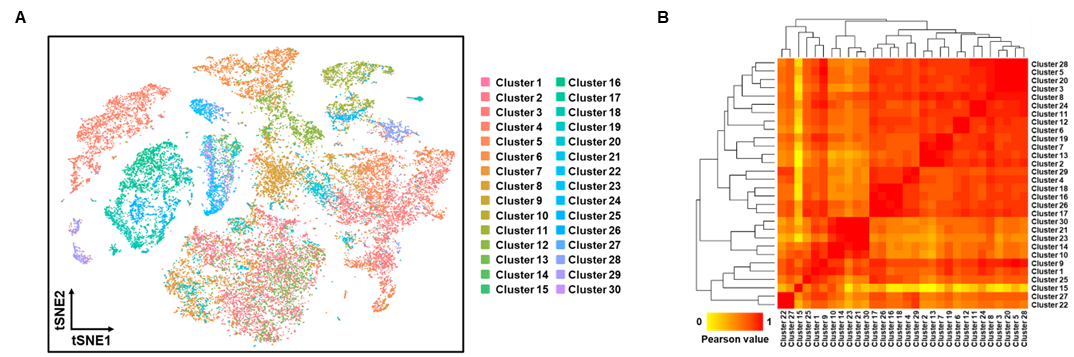


**Figure S2 Graph-based clustering using single-cell RNA sequencing**

(A) t-SNE visualization using graph-based clustering revealed 30 distinct populations.

(B) Color-coded correlation matrix of single-cell mRNA profiles.


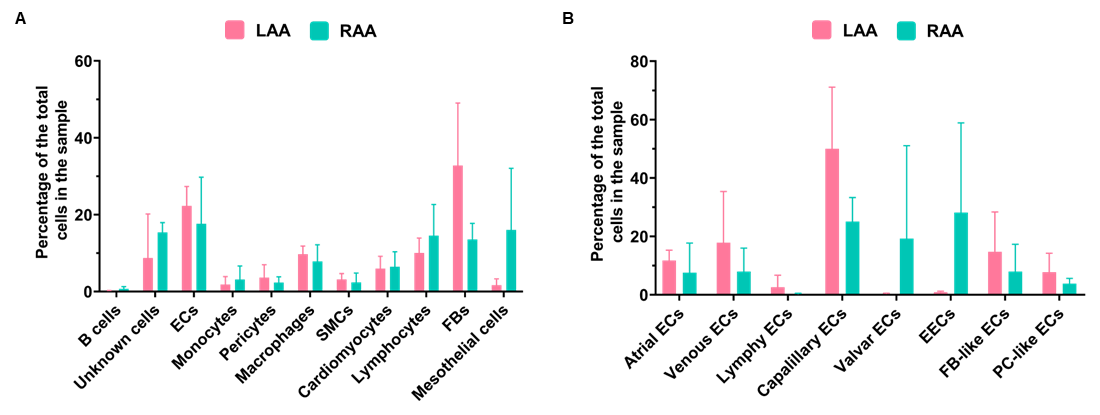


**Figure S3 Relative representation of cell clusters by sample in barplots**

(A) The relative proportions of cells types from individual samples of each donor within each cell type in all cells.

(B) The relative proportions of different EC clusters from individual samples of each donor.

Colors correspond to the cell type descriptions displayed in the panel above.


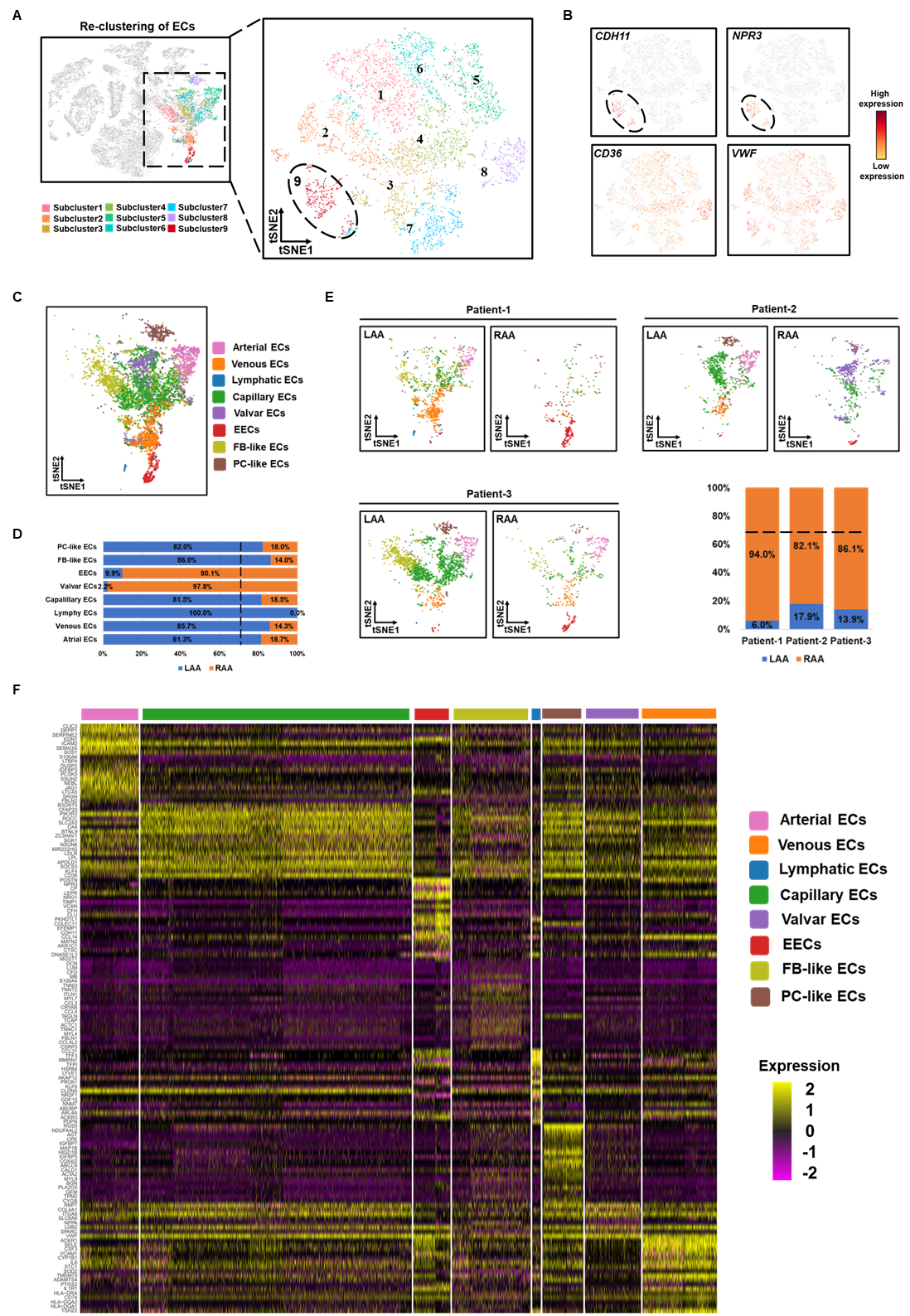


**Figure S4 Subclustering of endothelial cell populations**

(A) t-SNE plot representing nine observed ECs subclusters superimposed over the global t-SNE distribution. The dashed circle shows subcluster 9, which represents EECs.

(B) t-SNE plots showing the distribution in the expression of selected EEC markers (CDH11 and NPR3) that are enriched in the indicated subcluster 9 in (A) Expression is normalized on a color-coded scale.

(C) t-SNE plot representing identified subtypes of ECs.

(D) Composition of each subtype of EC is shown in the horizontal bar plot.

(E) Composition of EECs by tissue source of each donor is shown in the horizontal bar plot.

(F) Heat map showing the top 20 upregulated genes in each subtype of ECs. Cell populations were identified by the expression of known marker genes.

t-SNE, t-distributed stochastic neighbor embedding; ECs, endothelial cells, EECs, endocardial endothelial cells.


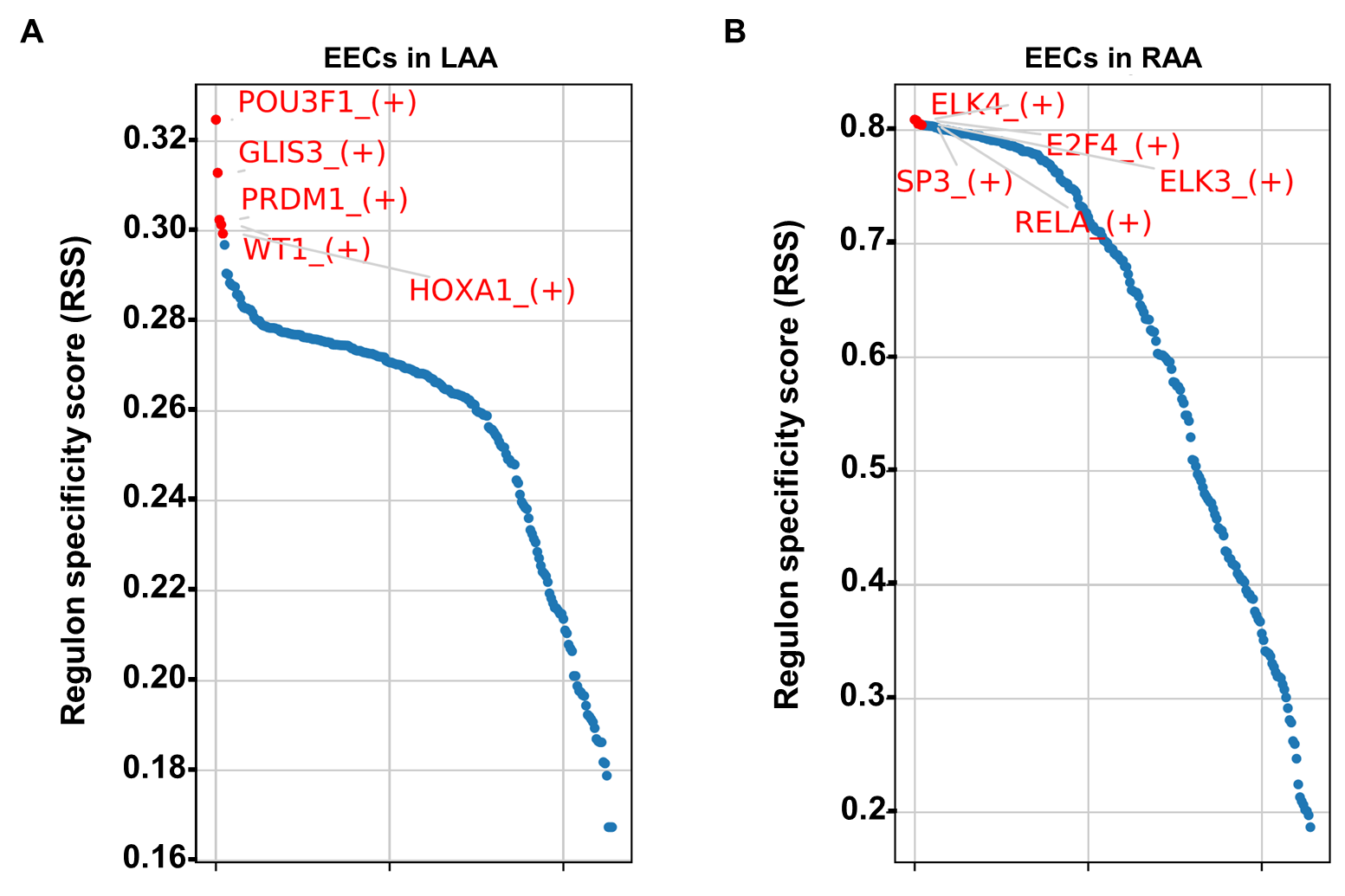


**Figure S5 Regulon specificity score for each of the EEC in LAA and RAA**

(A-B) Regulon specificity score for each of the EEC in LAA (A) and RAA (B) generated from regulon enrichment scores in each cell calculated using AUCell step of the SCENIC pipeline. Top 5 most specific regulons with high RSS in LAA and RAA separately are highlighted in red.

RRS, regulon specificity score


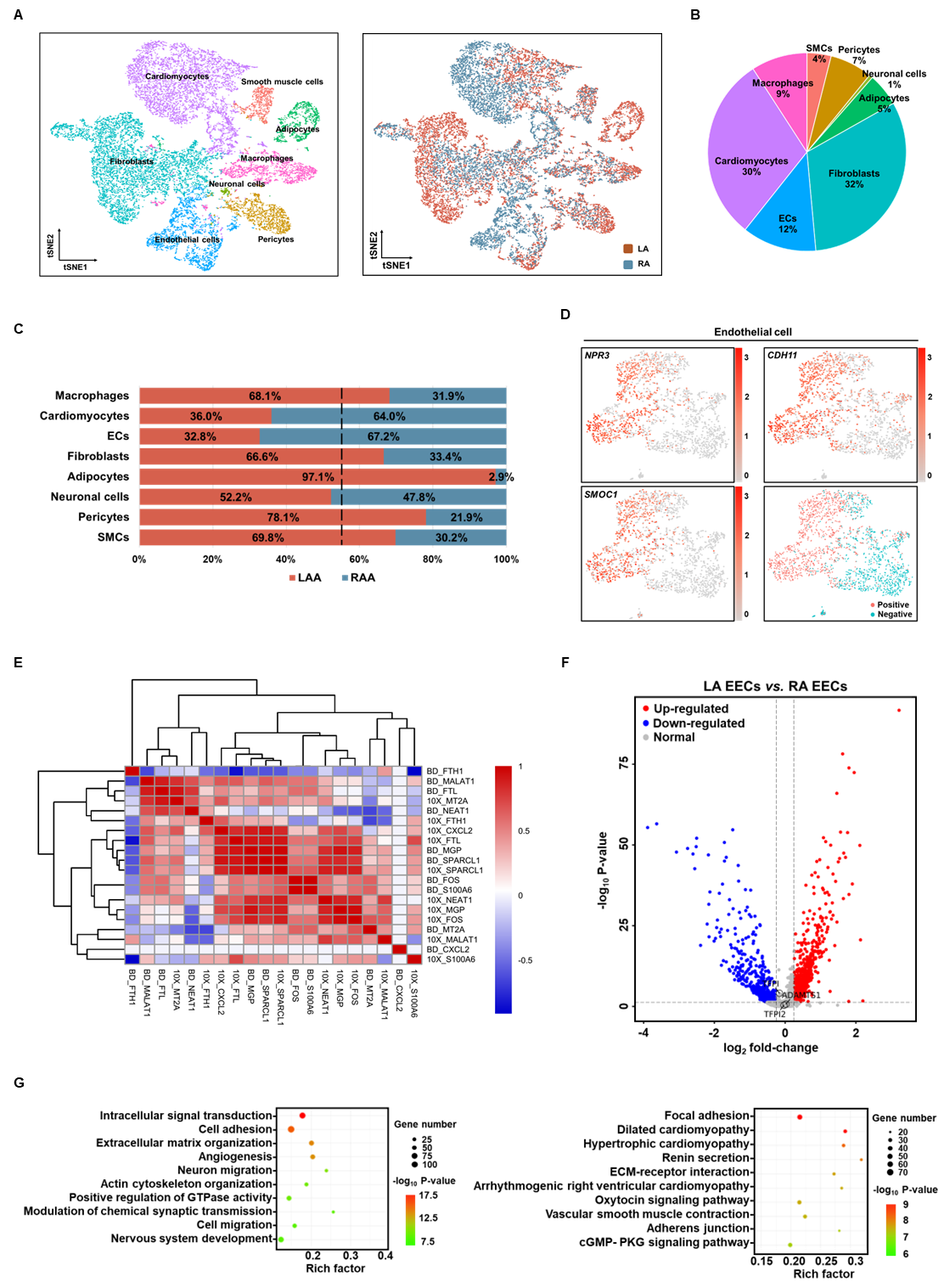


**Figure S6 Cardiac cell atlas and gene expression in atrial EECs in healthy male patients.**

(A) t-SNE plots of eight major cardiac cell types form healthy atrial samples. Each dot represents a single-cell and is colored according to the cell type designation (left) and sample source (right).

(B) Distribution of the identified cells according to cell type.

(C) The proportions of the identified cells in the left and right atrial tissue forming a healthy atrium.

(D) t-SNE plots are colored according to the expression of marker genes for EECs in cells from healthy males.

(E) Hierarchical clustering heatmap of Pearson correlation coefficients of top expressed genes between our scRNA-seq database and the referenced public database in identified EECs. Scale refers to Pearson correlation coefficient.

(F) Volcano plot illustrating differential gene expression in EECs from healthy left and right atrial tissue. Upregulated and downregulated genes in the EECs are colored red and blue, respectively.

(G) Dot plot shows the significantly enriched pathways by differential gene expression in EECs from healthy left and right atrial tissue.

EECs, endocardial endothelial cells.

**
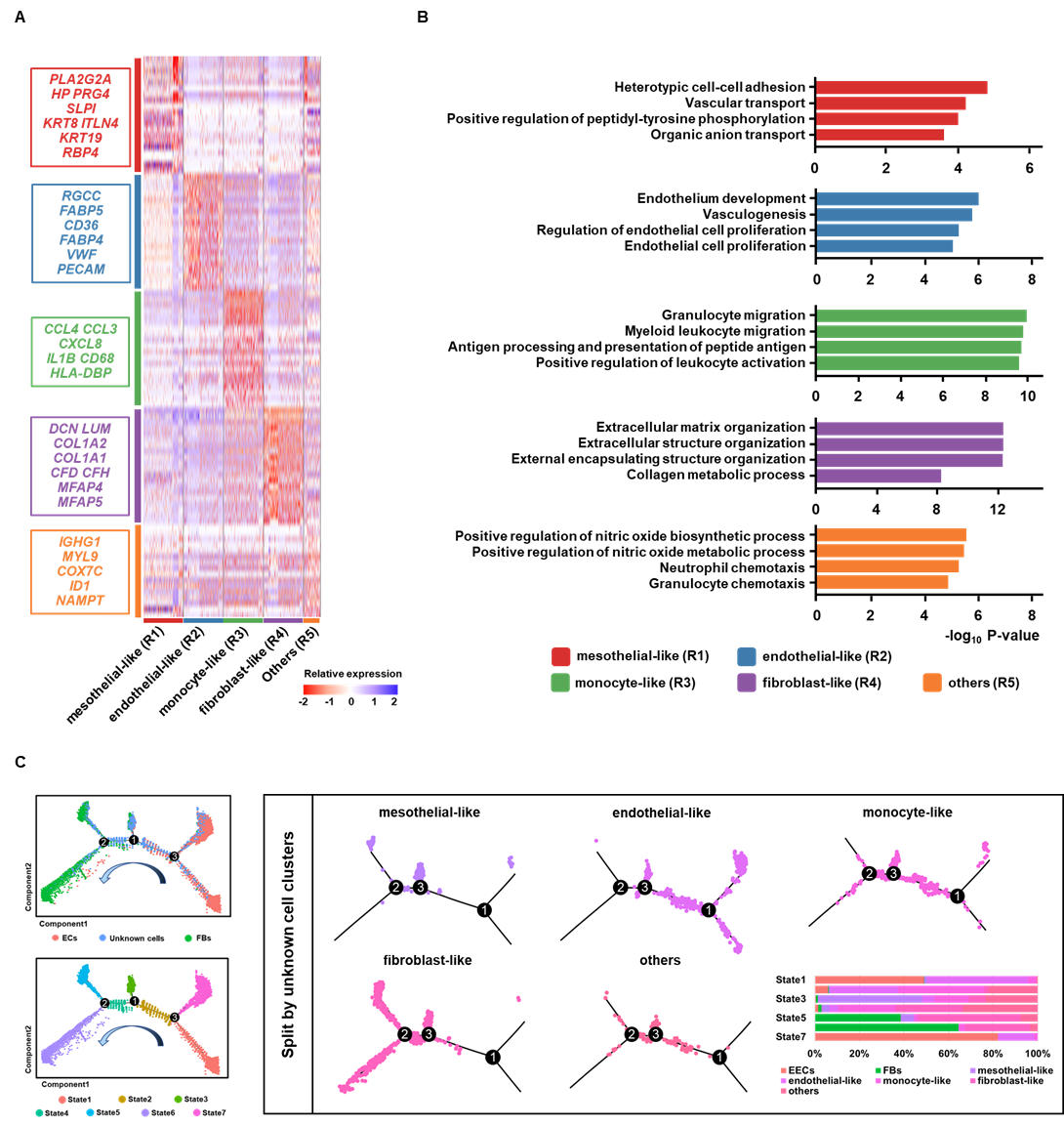
**

**Figure S7 Functional diversity and pseudotime-specific roles of unknown cell subtypes**

(A) Heat map showing the top 20 differentially expressed genes in five subclusters (R1-R5) among the unknown cells. Cell populations were identified by the expression of known marker genes.

(B) GO analysis showing enriched terms of differentially expressed genes for each indicated subcluster of unknown cells.

(C) Pseudotemporal order of EECs, unknown cells, and FBs presented according to cell clusters, cell state (left), and split view of trajectory analysis of unknown subclusters (right).

**
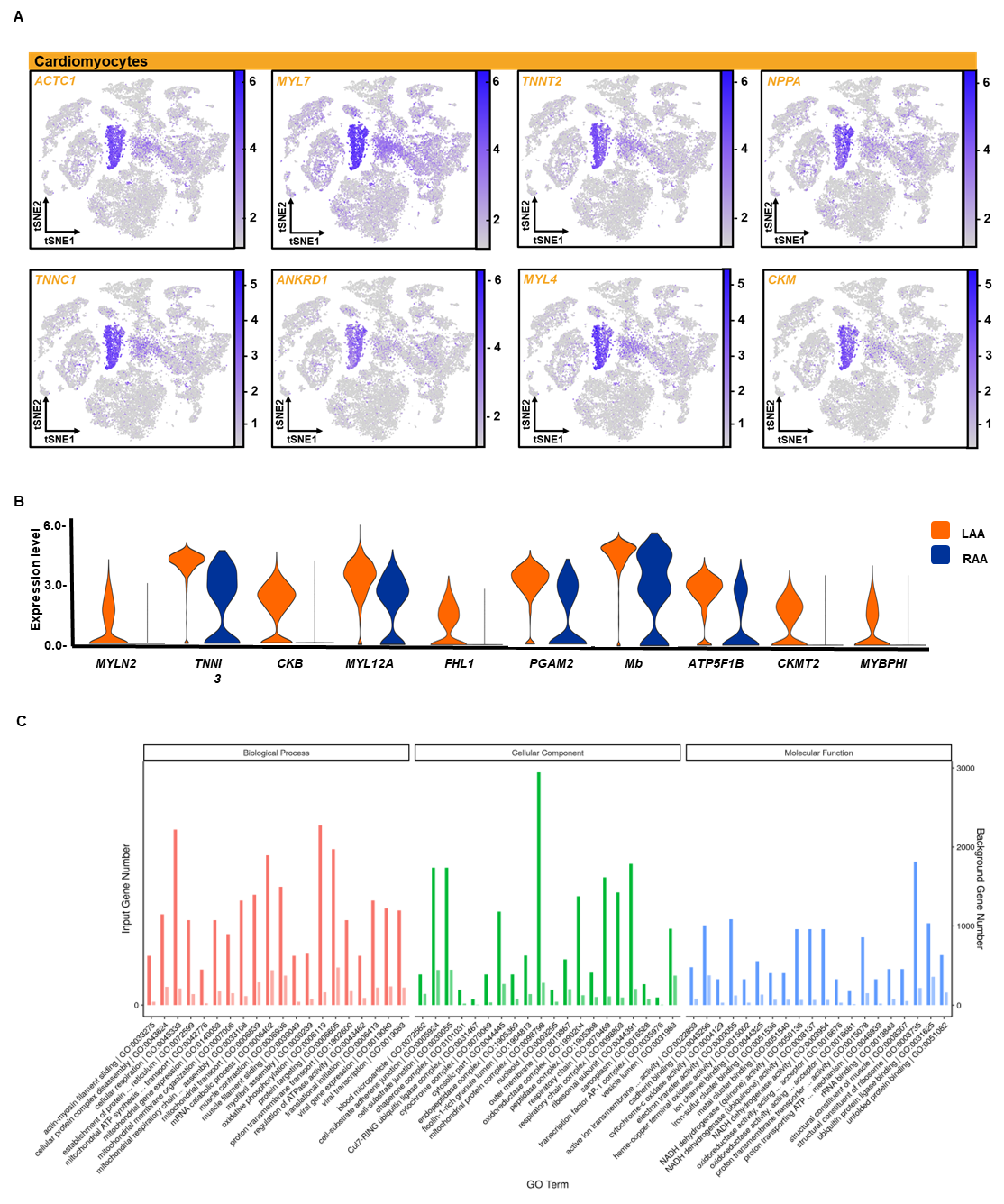
**

**Figure S8 Cardiomyocyte marker distribution**

(A) t-SNE projections are colored according to the expression of the cell-specific markers for CMs.

(B) Violin plots detailing the significant DEGs between LAA and RAA for CMs.

(C) Top 20 categories from GO enrichment analysis of DEGs between LAA and RAA for CMs.

t-SNE, t-distributed stochastic neighbor embedding; CMs, cardiomyocytes; DEGs, differentially expressed genes; LAA, left atrial appendage; RAA, right atrial appendage; GO, gene ontology.

**
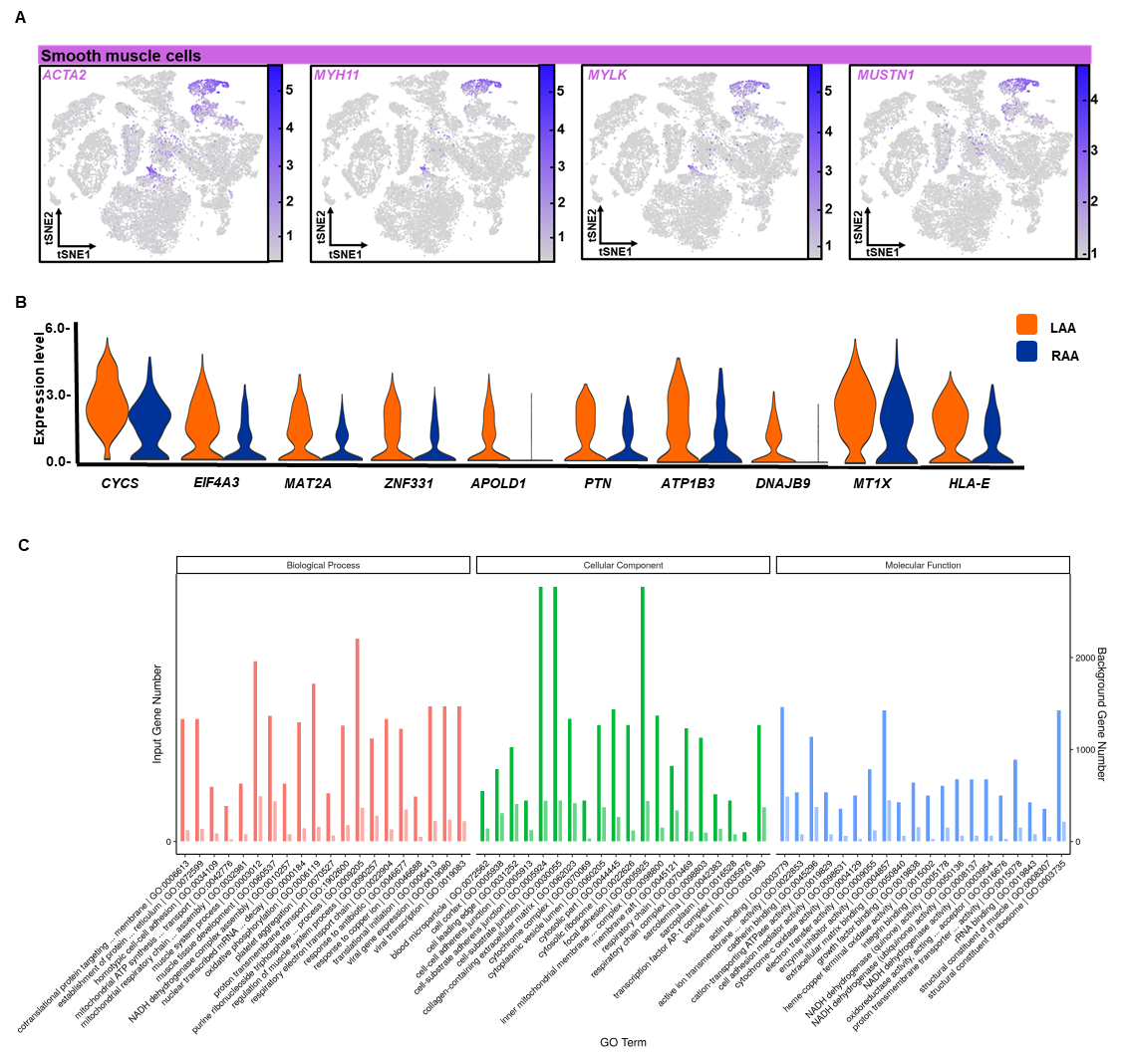
**

**Figure S9 Smooth muscle cells marker distribution**

(A) t-SNE projections are colored according to the expression of the cell-specific markers for SMCs.

(B) Violin plots detailing the significant DEGs between LAA and RAA for SMCs.

(C) Top 20 categories from GO enrichment analysis of DEGs between LAA and RAA for SMCs.

t-SNE, t-distributed stochastic neighbor embedding; SMCs, smooth muscle cells; DEGs, differentially expressed genes; LAA, left atrial appendage; RAA, right atrial appendage; GO, gene ontology.

**
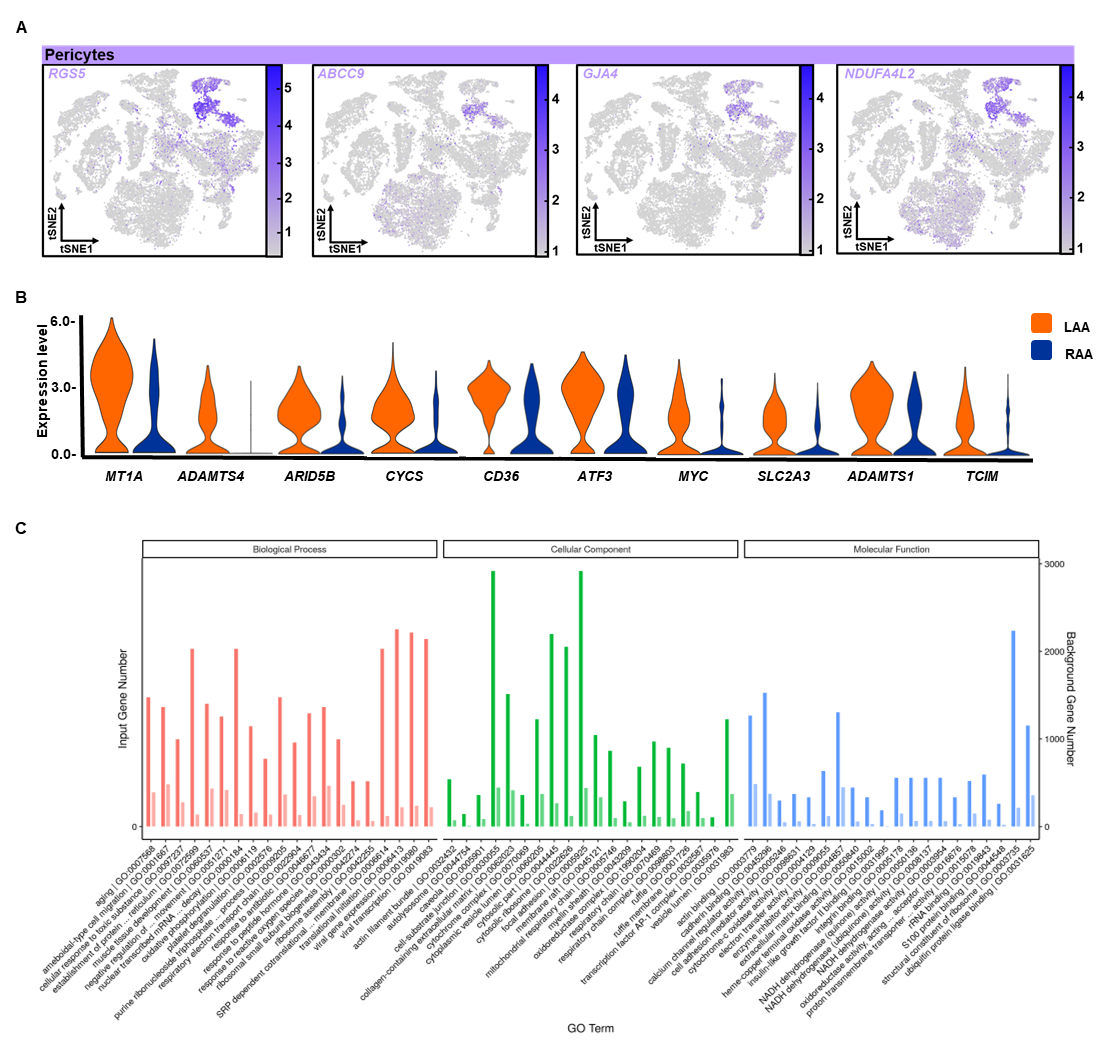
**

**Figure S10 Pericytes marker distribution**

(A) t-SNE projections are colored according to the expression of the cell-specific markers for pericytes.

(B) Violin plots detailing the significant DEGs between LAA and RAA for pericytes.

(C) Top 20 categories from GO enrichment analysis of DEGs between LAA and RAA for pericytes.

t-SNE, t-distributed stochastic neighbor embedding; DEGs, differentially expressed genes; LAA, left atrial appendage; RAA, right atrial appendage; GO, gene ontology.

**
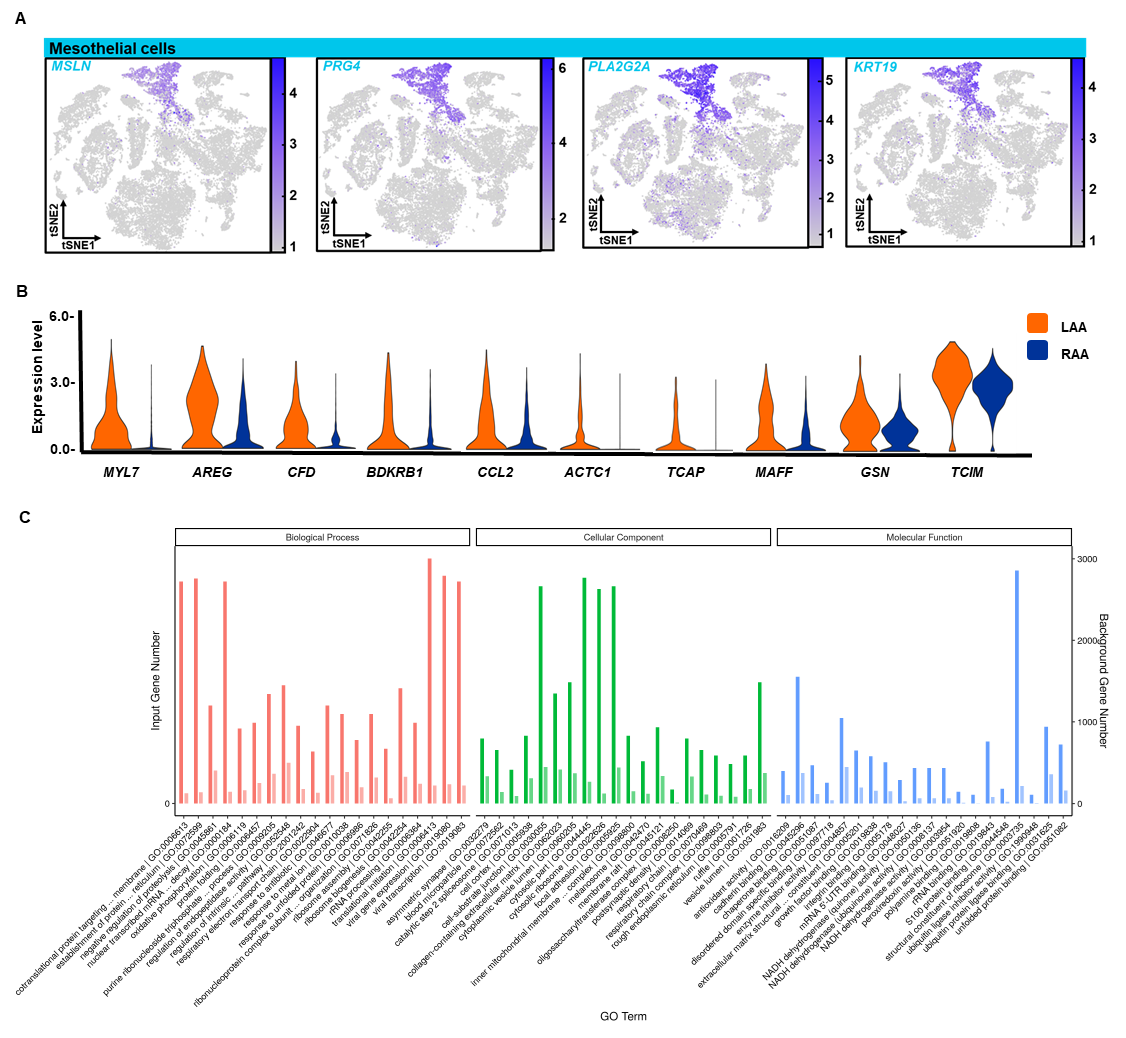
**

**Figure S11 Mesothelial cells marker distribution**

(A) t-SNE projections are colored according to the expression of the cell-specific markers for mesothelial cells.

(B) Violin plots detailing the significant DEGs between LAA and RAA for mesothelial cells. (C) Top 20 categories from GO enrichment analysis of DEGs between LAA and RAA for mesothelial cells.

t-SNE, t-distributed stochastic neighbor embedding; DEGs, differentially expressed genes; LAA, left atrial appendage; RAA, right atrial appendage; GO, gene ontology.


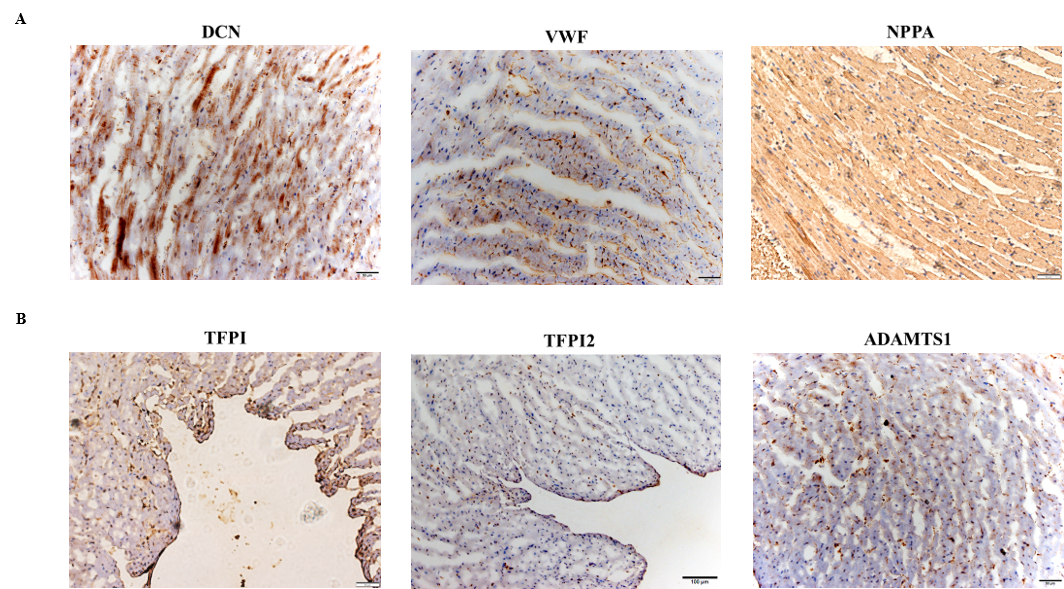


**Figure S12 Verification of cell markers and selective genes using immunohistochemistry**

(A) Examples of DCN (marker for FBs), VWF (marker for ECs), and NPPA (marker for CMs) expression on histological slides of AF mice. Scale bars: 50 µm.

(B) Examples of TFPI, TFPI2, and ADAMTS1 expression on histological slides of AF mice.

DCN, decorin; VWF, von Willebrand factor; NPPA, natriuretic peptide A; FBs, fibroblasts; ECs, endothelial cells; CMs, cardiomyocytes; TFPI, tissue factor pathway inhibitor; TFPI2, tissue factor pathway inhibitor 2; ADAMTS1, ADAM metallopeptidase with thrombospondin type 1 motif 1.


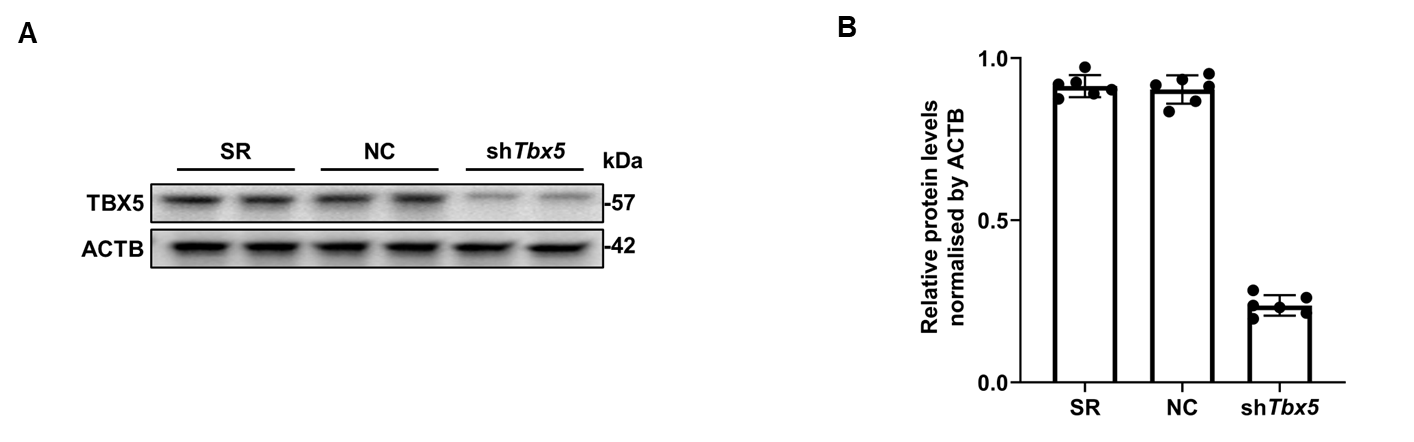


**Figure S13 The expression of TBX5 in EECs transfected with shRNA**

(A) Protein levels of TBX5 in EECs form SR mice (SR, n = 6), control-transfected EECs (NC, n = 6) and *Tbx5* shRNA-transfected EECs (sh*Tbx5*, n = 6) were analyzed using western blotting.

(B) Histogram depicting a significant reduction of TBX5 in sh*Tbx5*-transfected EECs compared to NC shRNA-transfected cells.

Tbx5, T-box transcription factor 5; EECs, endocardial endothelial cells; SR, sinus rhythm; NC, negative control.


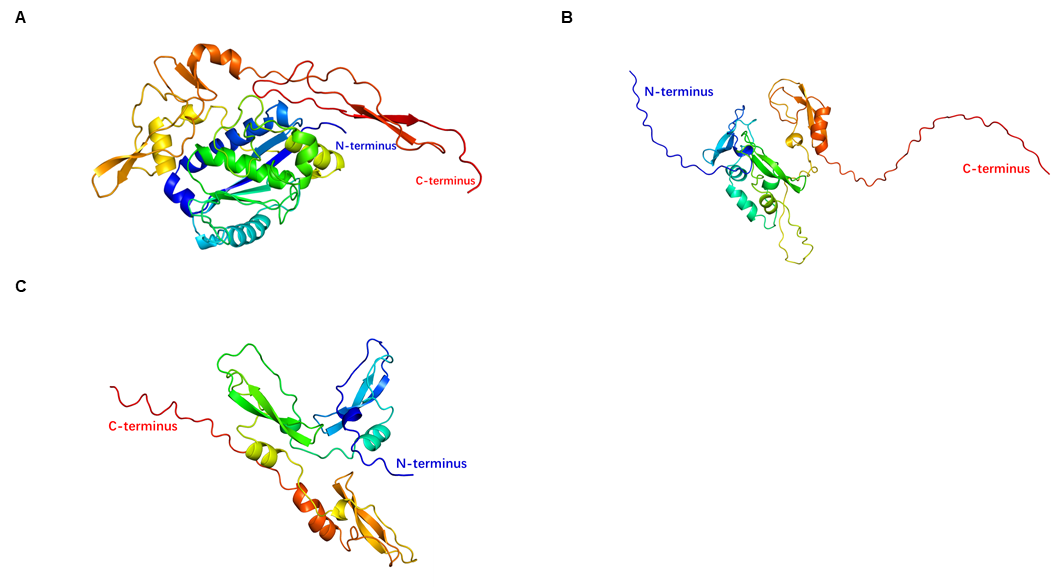


**Figure S14 Structure of predicted ADAMTS1, TFPI, and TFPI2**

(A–C) Structure of predicted ADAMTS1 (A), TFPI (B), and TFPI2 (C). The elements of the secondary structure (α-helices and β-sheets) are represented by different colors.


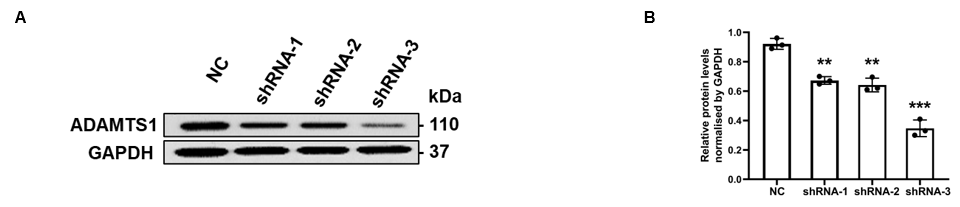


**Figure S15 ADAMTS1 protein ablation in endocardial endothelial cells transfected with shRNA**

(A) Protein levels of ADAMTS1 in control-transfected EECs (NC, n = 3) and ADAMTS1 shRNA-transfected EECs (shRNA-1, n = 3; shRNA-2, n = 3; shRNA-3, n = 3) were analyzed using western blotting.

(B) Histogram depicting a significant reduction of ADAMTS1 in shRNA-3-transfected EECs compared to NC shRNA-transfected cells.

EECs, endocardial endothelial cells; NC, negative control.

**3 Supplemental Tables**

**Table S1** **Demographic characteristics of the study subjects**

|  | **AF patient-1** | **AF patient-2** | **AF patient-3** |
| --- | --- | --- | --- |
| **Sex** | Male | Male | Male |
| **Age (yr)** | 62 | 51 | 66 |
| **Weight (kg)** | 63.4 | 72.5 | 74.9 |
| **Height (cm)** | 164.5 | 164 | 167 |
| **AF type** | Persistent AF | Persistent AF | Persistent AF |
| **Comorbidities** |  |  |  |
| **Hypertension** | × | × | √ |
| **Diabetes** | × | × | × |
| **Coronary artery disease** | × | × | × |
| **Myocardial infarction** | × | × | × |
| **Valve disease** | × | × | × |
| **Cardiomyopathy** | × | × | × |
| **Peripheral artery disease** | × | × | × |
| **Congestive heart failure** | × | × | × |
| **Chronic kidney disease** | × | × | × |
| **Chronic obstructive pulmonary disease** | × | × | × |
| **Cerebrovascular accident** | × | × | × |
| **Drugs on admission** |  |  |  |
| **Antihypertensives** | × | × | √ |
| **Statins** | × | × | × |
| **Antiplatelets** | × | × | × |
| **Non–vitamin K antagonist oral anticoagulant** | × | × | × |
| **Warfarin** | × | × | × |
| **Hear rate (beats/min)** | 83 | 90 | 88 |
| **Blood Pressure (mmHg)** |  |  |  |
| **Systolic** | 135 | 138 | 148 |
| **Diastolic** | 80 | 87 | 92 |
| **Echocardiographic findings** |  |  |  |
| **LAAP (cm)** | 4.7 | 4.5 | 4.1 |
| **LALR (cm)** | 5.1 | 4.5 | 4.6 |
| **IVS (cm)** | 1.2 | 1.2 | 1.4 |
| **PW Thick (cm)** | 1.1 | 1.1 | 1.2 |
| **LVEF (%)** | 63 | 62 | 60 |
| **Clinical laboratory results** |  |  |  |
| **Creat (mg/dl)** | 151.9 | 125.7 | 97.3 |
| **APTT (s)** | 38.1 | 29.9 | 35.9 |
| **PT (s)** | 12.2 | 15.1 | 12.3 |
| **INR** | 1.11 | 1.36 | 1.11 |

LAAP, left atrial anteroposterior diameter; LALR, left atrial left-right diameter; IVS, interventricular septum; PW Thick, posterior wall thicknesses; LVEF, left ventricular ejection fraction; Creat, creatinine; APTT, activates partial thromboplastin time; PT, prothrombin time; INR, international normalized ratio.

**Table S2** **Top 10 markers differentiating atrial appendage cell types**

| **Cell Type** | **Marker Genes** |
| --- | --- |
|  | \| gene \| p_val \| avg_log2FC \| gene \| p_val \| avg_log2FC \| \| --- \| --- \| --- \| --- \| --- \| --- \| |
| **Cardiomyocytes** | \| *ACTC1* \| 0 \| 5.30 \| *NPPA* \| 0 \| 4.79 \| \| --- \| --- \| --- \| --- \| --- \| --- \| \| *MYL7* \| 0 \| 5.24 \| *TNNC1* \| 0 \| 4.64 \| \| *MB* \| 0 \| 5.08 \| *ANKRD1* \| 0 \| 4.63 \| \| *TCAP* \| 0 \| 4.81 \| *COX6A2* \| 0 \| 4.62 \| \| *TNNT2* \| 0 \| 4.80 \| *MYL4* \| 0 \| 4.59 \| |
| **Fibroblasts** | \| *DCN* \| 0 \| 4.28 \| *GSN* \| 0 \| 3.41 \| \| --- \| --- \| --- \| --- \| --- \| --- \| \| *CFD* \| 0 \| 3.81 \| *SERPINF1* \| 0 \| 3.33 \| \| *LUM* \| 0 \| 3.56 \| *DPT* \| 0 \| 3.26 \| \| *FBLN1* \| 0 \| 3.44 \| *PTN* \| 0 \| 3.23 \| \| *MGP* \| 0 \| 3.42 \| *COL1A2* \| 0 \| 3.22 \| |
| **Endothelial cells** | \| *SELE* \| 0 \| 2.96 \| *FABP5* \| 0 \| 2.57 \| \| --- \| --- \| --- \| --- \| --- \| --- \| \| *C2CD4B* \| 0 \| 2.96 \| *FLT1* \| 0 \| 2.48 \| \| *VWF* \| 0 \| 2.72 \| *CAVIN2* \| 0 \| 2.42 \| \| *SLC9A3R2* \| 0 \| 2.67 \| *IFI27* \| 0 \| 2.40 \| \| *FABP4* \| 0 \| 2.58 \| *CAV1* \| 0 \| 2.36 \| |
| **Smooth muscle cells** | \| *TAGLN* \| 0 \| 3.75 \| *MYL9* \| 0 \| 3.10 \| \| --- \| --- \| --- \| --- \| --- \| --- \| \| *ACTA2* \| 0 \| 3.72 \| *MT1A* \| 0 \| 2.73 \| \| *MYH11* \| 0 \| 3.57 \| *RGS5* \| 0 \| 2.59 \| \| *MUSTN1* \| 0 \| 3.51 \| *C11orf96* \| 0 \| 2.55 \| \| *TPM2* \| 0 \| 3.41 \| *RERGL* \| 0 \| 2.48 \| |
| **Macrophages** | \| *CCL3* \| 0 \| 4.38 \| *CXCL8* \| 0 \| 3.52 \| \| --- \| --- \| --- \| --- \| --- \| --- \| \| *HLA-DRA* \| 0 \| 3.90 \| *CCL4* \| 0 \| 3.29 \| \| *CCL3L1* \| 0 \| 3.77 \| *CCL4L2* \| 0 \| 3.27 \| \| *IL1B* \| 0 \| 3.69 \| *C1QB* \| 0 \| 3.25 \| \| *C1QA* \| 0 \| 3.65 \| *HLA-DQA1* \| 0 \| 3.17 \| |
| **Mesothelial cells** | \| *MYL7* \| 0 \| 2.06 \| *DCN* \| 0 \| 1.26 \| \| --- \| --- \| --- \| --- \| --- \| --- \| \| *AREG* \| 0 \| 1.49 \| *MB* \| 0 \| 1.25 \| \| *CFD* \| 0 \| 1.36 \| *MYL4* \| 0 \| 1.20 \| \| *BDKRB1* \| 0 \| 1.32 \| *MT1G* \| 0 \| 1.17 \| \| *HBA2* \| 0 \| 1.28 \| *TNNI3* \| 0 \| 1.13 \| |
| **Pericytes** | \| *RGS5* \| 0 \| 4.01 \| *COX4I2* \| 0 \| 2.48 \| \| --- \| --- \| --- \| --- \| --- \| --- \| \| *NDUFA4L2* \| 0 \| 3.12 \| *ABCC9* \| 0 \| 2.42 \| \| *GJA4* \| 0 \| 2.68 \| *CPE* \| 0 \| 2.15 \| \| *HIGD1B* \| 0 \| 2.65 \| *MAP1B* \| 0 \| 2.09 \| \| *AGT* \| 0 \| 2.49 \| *NOTCH3* \| 0 \| 2.00 \| |
| **Lymphocytes** | \| *NKG7* \| 0 \| 4.23 \| *TUBA4A* \| 0 \| 3.13 \| \| --- \| --- \| --- \| --- \| --- \| --- \| \| *CCL5* \| 0 \| 3.81 \| *CXCR4* \| 0 \| 3.03 \| \| *GNLY* \| 0 \| 3.53 \| *CST7* \| 0 \| 2.98 \| \| *IL7R* \| 0 \| 3.39 \| *CD69* \| 0 \| 2.94 \| \| *GZMB* \| 0 \| 3.33 \| *PTPRC* \| 0 \| 2.93 \| |
| **Monocytes** | \| *S100A8* \| 0 \| 7.18 \| *ALOX5AP* \| 0 \| 4.10 \| \| --- \| --- \| --- \| --- \| --- \| --- \| \| *S100A9* \| 0 \| 5.83 \| *BASP1* \| 0 \| 4.02 \| \| *S100A12* \| 0 \| 4.71 \| *CCL20* \| 0 \| 3.86 \| \| *G0S2* \| 0 \| 4.20 \| *SAMSN1* \| 0 \| 3.69 \| \| *BCL2A1* \| 0 \| 4.11 \| *NAMPT* \| 0 \| 3.66 \| |
| **B cells** | \| *IGKC* \| 0 \| 7.41 \| *IGHG3* \| 0 \| 4.39 \| \| --- \| --- \| --- \| --- \| --- \| --- \| \| *IGHA1* \| 0 \| 5.77 \| *JCHAIN* \| 0 \| 4.36 \| \| *IGLC2* \| 0 \| 5.52 \| *IGHG4* \| 0 \| 4.18 \| \| *IGHG1* \| 0 \| 5.35 \| *IGHG2* \| 0 \| 3.94 \| \| *IGLC3* \| 0 \| 4.50 \| *CCR7* \| 0 \| 3.46 \| |
| **Unknown cells** | \| *HBB* \| 0 \| 2.71 \| *PLA2G2A* \| 0 \| 1.24 \| \| --- \| --- \| --- \| --- \| --- \| --- \| \| *HBA1* \| 0 \| 1.85 \| *KRT19* \| 0 \| 1.17 \| \| *HP* \| 0 \| 1.41 \| *C3* \| 0 \| 1.05 \| \| *ITLN1* \| 0 \| 1.36 \| *IGKC* \| 0 \| 0.98 \| \| *SLPI* \| 0 \| 1.33 \| *PRG4* \| 0 \| 0.94 \| |

**Table S3 Interaction pair of the best-scoring ADAMTS1-TFPI complex**

| Interaction Pair | Distance (Å) | Category | Types |
| --- | --- | --- | --- |
| ADAMTS1-TFPI |  |  |  |
| A:SER607:OG - B:GLU31:N | 2.25 | Hydrogen Bond | Conventional Hydrogen Bond |
| A:LYS592:NZ - B:ILE133:O | 2.38 | Hydrogen Bond | Conventional Hydrogen Bond |
| A:LYS548:NZ - B:GLU262:OE1 | 2.41 | Hydrogen Bond; Electrostatic | Salt Bridge |
| A:ARG604:CD - B:GLU34:OE2 | 2.54 | Hydrogen Bond | Carbon Hydrogen Bond |
| A:SER561:OG - B:LYS57:NZ | 2.61 | Hydrogen Bond | Conventional Hydrogen Bond |
| A:HIS553:CE1 - B:GLY132:O | 2.70 | Hydrogen Bond | Carbon Hydrogen Bond |
| A:GLY560:O - B:LYS57:NZ | 2.83 | Hydrogen Bond | Conventional Hydrogen Bond |
| A:ARG604:NH1 - B:GLU34:OE2 | 3.07 | Hydrogen Bond; Electrostatic | Salt Bridge |
| A:ARG606:NH1 - B:GLU34:O | 3.25 | Hydrogen Bond | Conventional Hydrogen Bond |
| A:ARG606:CD - B:GLU34:O | 3.67 | Hydrogen Bond | Carbon Hydrogen Bond |
| A:GLN472:NE2 - B:CYS226:SG | 3.75 | Hydrogen Bond | Conventional Hydrogen Bond |
| A:PHE558 - B:ILE133:CD1 | 3.83 | Hydrophobic | Pi-Sigma |
| A:LYS552:NZ - B:GLU254:OE1 | 4.26 | Electrostatic | Attractive Charge |
| A:ASP476:OD2 - B:ARG232:NH2 | 4.56 | Electrostatic | Attractive Charge |
| A:LYS552 - B:TRP216 | 4.61 | Hydrophobic | Pi-Alkyl |
| A:ARG604:NH2 - B:GLU34:OE1 | 4.61 | Electrostatic | Attractive Charge |
| A:ARG606:NH1 - B:ASP33:OD1 | 5.18 | Electrostatic | Attractive Charge |

**Table S4 Interaction pair of the best-scoring ADAMTS1-TFPI2 complex**

| Interaction Pair | Distance (Å) | Category | Types |
| --- | --- | --- | --- |
| ADAMTS1-TFPI2 |  |  |  |
| A:SER364:OG - B:GLN105:NE2 | 2.25 | Hydrogen Bond | Conventional Hydrogen Bond |
| A:ARG330:NH1 - B:HIS131 | 2.79 | Electrostatic | Pi-Cation |
| A:TYR581:OH - B:LEU50:N | 2.80 | Hydrogen Bond | Conventional Hydrogen Bond |
| A:TYR449:OH - B:ALA47:O | 2.98 | Hydrogen Bond | Conventional Hydrogen Bond |
| A:ARG330:NH1 - B:CYS130:SG | 3.22 | Hydrogen Bond | Conventional Hydrogen Bond |
| A:TYR449:OH - B:GLY67:CA | 3.25 | Hydrogen Bond | Carbon Hydrogen Bond |
| A:ASN457:ND2 - B:TYR42:OH | 3.34 | Hydrogen Bond | Conventional Hydrogen Bond |
| A:ASP374:OD2 - B:ARG134:NH1 | 3.89 | Hydrogen Bond; Electrostatic | Salt Bridge |
| A:ARG573 - B:TYR77 | 4.56 | Hydrophobic | Pi-Alkyl |
| A:MET583 - B:LEU48 | 4.76 | Hydrophobic | Alkyl |
| A:TYR449 - B:ARG46 | 4.82 | Hydrophobic | Pi-Alkyl |
| A:ARG330:NH1 - B:ASP103:OD2 | 4.86 | Electrostatic | Attractive Charge |
| A:PHE253 - B:LEU50 | 4.92 | Hydrophobic | Pi-Alkyl |
| A:TYR581 - B:LEU49 | 4.93 | Hydrophobic | Pi-Alkyl |
| A:PHE253 - B:LEU65 | 5.49 | Hydrophobic | Pi-Alkyl |
